# Templated misfolding of Tau by prion-like seeding along neuronal connections impairs neuronal network function and associated behavioral outcomes in Tau transgenic mice

**DOI:** 10.1007/s00401-015-1413-4

**Published:** 2015-04-11

**Authors:** Ilie-Cosmin Stancu, Bruno Vasconcelos, Laurence Ris, Peng Wang, Agnès Villers, Eve Peeraer, Arjan Buist, Dick Terwel, Peter Baatsen, Tutu Oyelami, Nathalie Pierrot, Cindy Casteels, Guy Bormans, Pascal Kienlen-Campard, Jean-Nöel Octave, Diederik Moechars, Ilse Dewachter

**Affiliations:** Alzheimer Dementia Group, Institute of Neuroscience, Catholic University of Louvain, 1200 Brussels, Belgium; Department of Neurosciences, University of Mons, 7000 Mons, Belgium; Department of Neuroscience, Janssen Research and Development, A Division of Janssen Pharmaceutica NV, 2340 Beerse, Belgium; reMYND nv, Gaston Geenslaan 1, 3001 Leuven, Belgium; VIB11 vzw Center for the Biology of Disease, KU Leuven, 3000 Leuven, Belgium; MoSAIC-Molecular Small Animal Imaging Centre, KU Leuven, 3000 Leuven, Belgium

**Keywords:** AD, Tauopathies, Tau, Prion-like propagation, Tau-pathology, Neuronal network, Synaptic, Cognition, Motoric deficits, TauP301 heterogeneity

## Abstract

Prion-like seeding and propagation of Tau-pathology have been demonstrated experimentally and may underlie the stereotyped progression of neurodegenerative Tauopathies. However, the involvement of templated misfolding of Tau in neuronal network dysfunction and behavioral outcomes remains to be explored in detail. Here we analyzed the repercussions of prion-like spreading of Tau-pathology via neuronal connections on neuronal network function in TauP301S transgenic mice. Spontaneous and GABA_A_R-antagonist-induced neuronal network activity were affected following templated Tau-misfolding using synthetic preformed Tau fibrils in cultured primary neurons. Electrophysiological analysis in organotypic hippocampal slices of Tau transgenic mice demonstrated impaired synaptic transmission and impaired long-term potentiation following Tau-seed induced Tau-aggregation. Intracerebral injection of Tau-seeds in TauP301S mice, caused prion-like spreading of Tau-pathology through functionally connected neuroanatomical pathways. Electrophysiological analysis revealed impaired synaptic plasticity in hippocampal CA1 region 6 months after Tau-seeding in entorhinal cortex (EC). Furthermore, templated Tau aggregation impaired cognitive function, measured in the object recognition test 6 months post-seeding. In contrast, Tau-seeding in basal ganglia and subsequent spreading through functionally connected neuronal networks involved in motor control, resulted in motoric deficits reflected in clasping and impaired inverted grid hanging, not significantly affected following Tau-seeding in EC. Immunostaining, biochemical and electron microscopic analysis in the different models suggested early pathological forms of Tau, including Tau-oligomers, rather than fully mature neurofibrillary tangles (NFTs) as culprits of neuronal dysfunction. We here demonstrate for the first time using in vitro, ex vivo and in vivo models, that prion-like spreading of Tau-misfolding by Tau seeds, along unique neuronal connections, causes neuronal network dysfunction and associated behavioral dysfunction. Our data highlight the potential relevance of this mechanism in the symptomatic progression in Tauopathies. We furthermore demonstrate that the initial site of Tau-seeding thereby determines the behavioral outcome, potentially underlying the observed heterogeneity in (familial) Tauopathies, including in TauP301 mutants.

**Electronic supplementary material:**

The online version of this article (doi:10.1007/s00401-015-1413-4) contains supplementary material, which is available to authorized users.

## Introduction

Tauopathies are a diverse group of neurodegenerative diseases, characterized by the presence of Tau aggregates composed of misfolded hyperphosphorylated Tau [7, 41, 62]. In Tauopathies, symptoms correlate strongly with the regional distribution of Tau-aggregates in the brain. In Alzheimer’s Disease, the most common Tauopathy, Tau aggregate formation progresses in a stereotypic pattern, along functionally connected neuroanatomical pathways, used for staging disease progression [2, 6, 34, 59]. In AD, Tau pathology starts in the entorhinal cortex (EC), progresses to the limbic regions such as subiculum, hippocampal cornu ammonis (CA) and amygdala (stages III and IV), and finally involves neocortical areas (stages V and IV), according to the Braak and Braak stages [6, 59]. Defects in neuronal function and neuronal loss of the affected or connected neurons are believed to contribute to the disease symptoms by affecting functionally critical neuronal networks, while the mechanism remains poorly understood [6, 15, 24, 58, 74].

Accumulating evidence indicates that Tau and related proteins linked to neurodegenerative proteinopathies display prion-like properties [12–14, 19, 22, 25, 26, 28, 32, 35, 37, 72]. In vitro studies have demonstrated that extracellular misfolded Tau can be taken up by cultured cells, can seed aggregation of endogenous soluble Tau and can propagate a fibrillar, misfolded state between co-cultured cells [20, 26, 32, 53]. Injections of brain lysates from neurofibrillary tangle (NFT) bearing transgenic mice into wild type Tau-expressing transgenic mice that do not develop NFTs, induce Tau pathology that spreads to brain regions remote from the injection site by connectivity rather than proximity [1, 13]. This has been recapitulated with brain extracts from patients with different Tauopathies, mimicking distinct forms of Tau-aggregation—in a cell-type and region specific way-reminiscent for the respective Tauopathies [5, 12]. Injection of pre-aggregated synthetic full length Tau and Tau fragments results in seeding and spreading of Tau-pathology in cells and in Tau transgenic mice, demonstrating that misfolded Tau per se, and not a different factor in the brain extracts, is sufficient to induce Tau-aggregation [35]. Induction of Tau-aggregation by misfolded Tau has been demonstrated in non-transgenic mice [40], albeit at later age and to a more limited extent. Indefinite and stable maintenance of unique conformations—“strains”—in vivo that link structure to patterns of pathology was recently demonstrated, further corroborating prion-like properties of misfolded Tau [5, 53]. Although increasing dysfunction in neuronal networks is believed to explain the progression of behavioral disease symptoms, including memory loss [22, 28, 53, 58, 59], it remains to be demonstrated whether this can be caused by prion-like spreading of Tau-pathology. This is not a trivial question, as previous reports have indicated that NFTs per se may not disturb neuronal function and could be an off-pathway disease side effect [30, 38, 48, 52, 54, 61, 65, 66, 69].

In this work, we have addressed this question using in vitro, ex vivo and in vivo models with induction and spreading of Tau-pathology. Seeding of Tau pathology was shown to impair neuronal network function in primary neuronal cultures and in organotypic hippocampal slices. Furthermore, Tau-seeding caused prion-like spreading of Tau-aggregation through functionally connected neuronal networks and neuronal network dysfunction in TauP301S transgenic mice, leading to either cognitive or motoric deficits, depending on the initial site of Tau-seeding. Our data furthermore point to early pathological forms of Tau, including Tau oligomers, rather than somatic NFTs as culprits for the functional deficits.

## Materials and methods

### Animals

Transgenic TauP301S mice (PS19) [73] expressing human Tau-P301S (1N4R), driven by the mouse prion protein promoter were backcrossed to C57B6 and used in this study. TauP301S mice bred and used in our lab, develop a neurodegenerative phenotype similar as previously reported, at ~10–11 months [35, 63, 73]. Stereotactic injections were performed at 3 months and age-matched littermates were used for analysis at different time points post-injection. All experiments were performed in compliance with protocols approved by the UCLouvain Ethical Committee for Animal Welfare.

### Tau aggregation assays

*Generation of Tau*-*PFFs from recombinant Tau* Tau-PFFs (synthetic preformed fibrils) or Tau-seeds were generated as described [26, 35]. Briefly, truncated human Tau fragments (WT-Tau/P301L-Tau) containing the four repeat domain [K18; Q244-E372 (4RTau)], N-terminally Myc-tagged were produced in *Escherichia coli*. Tau-PFFs were obtained by incubation of Tau fragments (66 μM) at 37 °C for 5 days in the presence of heparin (133 μM) in 100 mM ammonium acetate buffer (pH 7.0). Following centrifugation (100,000*g*, 1 h, 4 °C), the pellet was resuspended in the same buffer (333 µM final) and sonicated before use. Tau fibrilization was confirmed using ThioT assay (Sigma-Aldrich, St. Louis, MO, USA) and immunoblotting. *In vitro Tau aggregation assay in HEK293 cells* PFFs induced Tau-aggregation in vitro was essentially performed as previously [26]. Sonicated Tau-seeds (10 μM) were added to Bio-PORTER single use tubes (AMS Biotechnology, Milton, UK) and added to optiMEM-washed transiently transfected HEK293 (QBI) cells, 24 h post-transfection with Tau (P301L or WT). *In vitro Tau*-*aggregation assay in primary neurons* Primary cortical neuronal cultures (PNC) were generated as described previously [55], from P0 heterozygous TauP301S pups or non-transgenic (WT) littermates. Tau-seeds (10 nM) were added at DIV3 and DIV6, and primary neurons were used for calcium imaging at DIV13 and subsequently extracted or fixed for further analysis. *Ex*-*vivo Tau*-*aggregation assay in organotypic hippocampal slices* Organotypic hippocampal slices (OC) were generated using previously described protocols [21, 64]. Briefly, hippocampal slices were generated at P6 from heterozygous TauP301S mice and non-transgenic littermates. Tau-seeds (1 µL; 333 µM) were gently added on top of hippocampal slices at DIV3 and DIV6, and slices were analyzed electrophysiologically, biochemically and immunohistologically 10 days after seeding. *Tau*-*seeded Tau*-*aggregation in vivo* Sonicated pre-aggregated Tau-PFFs (5 µL; 333 µM) or vehicle without seeds (5 µL) were injected in 3 months old mice. Stereotactic injections were performed in the hippocampal CA1 region (A/P, −2.0; L, +1.4; D/V, −1.2), frontal cortex (A/P, +2.0; L, +1.4; D/V, −1.0), in entorhinal cortex (A/P, −4.8; L, −3.0; D/V, −3.7) and substantia nigra (A/P, −4.8; P/A, angle 16°; L, −1.1; D/V, −4.7) millimeter relative to bregma [45], using a 10 µL Hamilton syringe at a speed of 1 μL per min.

### Biochemical analysis

For immunoblotting analysis brain regions were dissected and snap-frozen in liquid nitrogen, and subsequently differential extraction of total homogenates, sarkosyl soluble and sarkosyl insoluble fractions was performed as previously described [67, 68] and in supplemental data. Similar extraction procedures were used for primary neurons and organotypic cultures as described in supplemental data. Proteins were quantified using BCA Protein Assay kit (Thermo Fisher Scientific, Waltham, MA, USA). For dot blotting equal amounts of total homogenates were spotted on a nitrocellulose membrane and subsequently immunoblotted using T22 [ABN454 Anti-Tau (T22) oligomeric antibody; EMD Millipore], against oligomeric Tau [39]. Analysis under non-denaturating and non-reducing conditions was performed on 4-16 % Bis-Tris Native Page. For immunoblotting equal amounts of proteins were loaded on precasted gels [4–12 % (MOPS), 8 % Tris–glycine gel (Invitrogen, Life Technologies, Carlsbad, CA, USA)]. Immunoblotting was performed with anti-Tau P-S202/T205 (AT8; Thermo Fisher Scientific, Waltham, MA, USA), with anti-Tau P-S396/S404 antibody (AD2; Bio-Rad Laboratories Inc., Hercules, CA, USA), or with anti-total Tau antibody (HT7; Thermo Fisher Scientific, Waltham, MA, USA) and developed using ECL kit (PerkinElmer, Waltham, MA, USA).

### Immunohistological and immunocytological analysis

Immunohistological analysis was performed as described previously [16, 17, 36, 49, 63]. Following transcardial flushing (2 min), brains were dissected and immersion fixed in 4 % paraformaldehyde in PBS for 24 h at 4 °C. Sagittal sections (40 µm) were cut on a vibrating HM650V microtome (Thermo Fisher Scientific, Waltham, MA, USA). Immunohistochemistry (IHC) with anti-Tau P-S202/T205 (AT8), anti-Tau P-S212/T214 (AT100) and anti-conformational specific Tau (MC1, Peter Davies) was done on 40 µm sagittal vibratome sections using appropriate Alexa coupled secondary antibodies (Invitrogen, Life Technologies, Carlsbad, CA, USA). Staining with Thioflavin S (ThioS; Sigma-Aldrich, St. Louis, MO, USA) and Gallyas silver staining were performed as previously described [63]. Image acquisition was performed using a digital inverted fluorescence microscope EVOS-xl microscope (Life Technologies, Carlsbad, CA, USA), using a 4×, 10×, 20× or 40× lens, and standard light microscope. Image analysis was done using Image J (National Institutes of Health). Heat maps were generated using the HeatMap Histogram plugin for Image J. Briefly, the overview images of AT8 staining of a well-identified section of different mice (*n* ≥ 3 per group) were grouped. Stacked images representing averaged intensities were generated using the Image J stacking tool with the average intensities outcome option. Finally, a Gaussian Blur filter of 5.0 was applied. Immunocytochemistry (ICC) and IHC on HEK293 cells, primary neurons and organotypic cultures were performed similarly. Fixation was performed standard or under stringent extraction of soluble Tau (supplemental material). All other chemicals were from Sigma-Aldrich (Sigma-Aldrich, St. Louis, MO, USA).

### Neuronal network activity-synchronized cytosolic calcium oscillations

Primary neurons from P0, TauP301S mice and non-transgenic littermates were used for calcium imaging, using incubation with fura-2 acetoxymethylester (Fura-2 AM; Calbiochem, Camarillo, CA, USA) 2 μM (final) in Krebs-HEPES buffer (10 mM HEPES, 135 mM NaCl, 6 mM KCl, 2 mM CaCl_2_, 1.2 mM MgCl_2_, 10 mM glucose, pH 7.4) for 30 min at RT. Coverslips were then washed in Krebs–HEPES buffer and mounted in a heated (37 °C) microscope chamber with 600 µL of buffer [44, 55]. After recording of basal spontaneous calcium oscillations, picrotoxin (PTX; Sigma-Aldrich, St. Louis, MO, USA) was added to the neurons to a final concentration of 100 µM for 3 min. Cells were alternately excited (1 or 2 Hz) at 340 and 380 nm for 100 ms using a Lambda DG-4 Ultra High Speed Wavelength Switcher (Sutter Instrument, Novato, CA, USA) coupled to a Zeiss Axiovert 200 M inverted microscope (20x fluorescence objective) (Carl Zeiss AG, Oberkochen, DE). Images were acquired with a Zeiss Axiocam camera coupled to a 510 nm emission filter and analyzed with the AxioVision software. Calcium concentration was evaluated from the ratio of fluorescence emission intensities excited at the two wavelengths, i.e. the ratio of F340/F380. Changes in the intracellular calcium fluorescence were expressed as Δ*F*/*F*_0_ to represent the changes in the cytosolic calcium concentrations, relative to the resting fluorescence value *F*_0_. Calcium oscillations were defined as variations of more than 10 % from *F*_0_, occurring synchronously in several cells of the field.

### Functional analysis

*Electrophysiology* Electrophysiological analysis in organotypic hippocampal slices was performed on non-seeded (vehicle) or Tau-seeded cultured hippocampal slices (at DIV3 and DIV6) from WT or TauP301S P6 pups and analyzed after the indicated time post-seeding (2, 4, 10 days). Cultures were directly placed into the recording chamber and slices were kept in interface at 28 °C for 30 min before recordings. Electrophysiological analysis in acute hippocampal slices derived from seeded and non-seeded mice was performed essentially as described previously [17, 63]. Briefly, hippocampus was dissected and cut in 450 µm-thick slices with a tissue chopper. The slices were transferred into the recording chamber and kept in interface at 28 °C for 90 min. *Electrophysiological recordings* Hippocampal slices were perfused with artificial cerebrospinal fluid (ACSF) with the following composition: 124 mM NaCl, 5 mM KCl, 26 mM NaHCO_3_, 1.24 mM NaH_2_PO_4_, 2.5 mM CaCl_2_, 1.3 mM MgSO_4_, 10 mM glucose, bubbled with a mixture of 95 % O_2_ and 5 % CO_2_. The perfusion rate of ACSF was 1 mL/min. A bipolar twisted nickel-chrome electrode (50 µm diameter) was used to stimulate Schaffer’s collaterals. Extracellular field excitatory postsynaptic potentials (fEPSP) for acute slices or population spikes (PS) for organotypic slices were recorded in the stratum radiatum of the CA1 region with low resistance (2–5 MΩ) glass microelectrodes filled with ACSF. The slope of the fEPSP or the amplitude of PS was measured on the average of four consecutive responses. LTP was induced by applying one train of high-frequency stimulation (100 Hz, 1 s). Stimulation, data acquisition and analysis were performed using the WinLTP program 28 (website: http://www.winltp.com). For each slice, the fEPSP slopes or PS amplitudes were normalized with respect to the mean slope of the fEPSPs or the mean amplitude of the PS recorded during the 30 min period preceding induction of LTP. Statistical analysis of the data was performed using one-way ANOVA or Student’s *t* test in SigmaPlot 12.0 software (Systat Software Inc, Chicago, IL, USA). Data were expressed as mean ± SEM, and differences with *p* < 0.05 were considered significant. *FDG-PET analysis* Brain glucose metabolism was assessed using [^18^F]FDG (FDG) as described in detail in supplemental data [9].

### Behavioral testing

*The object recognition task* The object recognition task was essentially performed as previously described [17, 51]. Briefly, following 10 min habituation to the open field box (60 × 60 × 50 cm), the mice were submitted the next day to a 10 min acquisition trial. During this trial, mice were placed in the open field in the presence of object A, and the time spent exploring object A was measured. During a 10 min retention trial performed 1 h later, a novel object B was added to the arena. The recognition index (RI), defined as the ratio of the time spent exploring the novel object B over the time spent exploring both objects [(*t*B/(*t*A + *t*B)) × 100] was used to measure nonspatial memory [17]. *Clasping scoring* Scoring of clasping was performed using a scale between 5 and 1, with clasping score 5 representing no clasping (normal), score 4 representing initial signs of clasping, score 3 for intermediate clasping, score 2 for strong clasping, and score 1 representing very severe (maximal) clasping. Scores between 1 and 5 were assigned by an experimentator blinded for the experimental group. *Inverted grid hanging task* The inverted grid hanging test was used to assess the ability to grasp an elevated horizontal grid and to remain suspended for 2 min. The grid (40 cm × 20 cm/0.5 cm meshes) was positioned 50 cm above a flat, soft surface and the latency for the animal to drop off was measured.

### Immunogold electron microscopy

Protein extracts from mouse brains, PNC, OC and HEK293 cells were applied on 300-mesh carbon-coated grids in drops of 3 µL for 5 min, blotted on a filter paper and air-dried before the immunogold procedure. Grids were blocked in PBS, 0.1 % cold water fish gelatin (PBS-CWFG) for 5 min before incubating with PBS-CWFG diluted primary antibodies (HT7 1:50, AT8 1:50, AT100 1:50) for 90 min at RT. After washing 5 times (2 min) in PBS-CWFG, grids were incubated for 60 min with secondary antibody (goat anti-mouse IgG) labeled with 10-nm gold particles (Aurion) diluted 1:30 in PBS-CWFG. Following washes in PBS and dH2O, grids were negatively stained with 2 % uranyl acetate for 1 min. The grids were examined with a JEOL JEM1400 transmission electron microscopy equipped with a Olympus SIS Quemesa 11 Mpxl camera, and images were taken at magnifications of 20× and 30× k (resp. pixel size = 0.72 and 0.48 nm). Immuno-gold electron-microscopy on brain slices is described in detail in supplemental data.

### Statistical analysis

Statistical analysis was performed using GraphPad Prism 5.0 (GraphPad Software, San Diego, CA, USA) using one-way analysis of variance (ANOVA) followed by Dunnett’s post hoc test and Student’s *t* test. Data were expressed as mean ± SEM and differences were considered significant when *p* values <0.05.

## Results

### Synthetic Tau-fibrils induce Tau-aggregation and altered neuronal network activity in primary neuronal cultures

Tau seeds extracted from brains of Tau transgenic mice with Tau pathology, from patients with different forms of Tauopathies or pre-aggregated synthetic Tau and Tau-fragments [12, 13, 35] induce Tau-aggregation in cultured cells and in vivo [19, 22, 26, 28, 53]. In this work, we used synthetic pre-aggregated Tau fragments, encompassing the four repeat domain of TauP301L, further referred to as “Tau-seeds”, to analyze the functional repercussions of Tau-aggregation in different in vitro, ex vivo and in vivo models. We first acquired and characterized Tau-seed induced Tau-aggregation in HEK293 cells, by administration of Tau-seeds to transfected TauP301L HEK293 cells using BioPORTER as previously shown [26]. Biochemical analysis demonstrated the presence of detergent insoluble (Triton-insoluble (TX-100) and sarkosyl insoluble) Tau-aggregates phosphorylated at S202/T205 (AT8) (Fig. S1a) following Tau-seeding while absent in non-seeded cells. Tau aggregation was further confirmed by immunocytochemical (ICC) analysis using a stringent extraction protocol with TX-100, extracting soluble forms of Tau from the cells, while only aggregated forms of Tau remained detectable. Staining with anti-Tau P-S202/T205 antibody (AT8) demonstrated intense staining of Tau aggregates in seeded cells, absent in non-seeded cells (Fig. S1a). Since the AT8 epitope is situated outside of the Tau-seeding fragment, this demonstrates aggregation of full length Tau and not accumulation of Tau-seeds in the cells. To determine optimal conditions for Tau-aggregation, we used different combinations of wild type Tau and mutant TauP301L forms as inducer and receiver. Immunocytological and alpha-LISA analysis demonstrated that wild type Tau was markedly less prone to aggregate than TauP301L (recipient), when seeded with either wild type or P301 mutated pre-aggregated Tau-seeds (inducer) (Fig. S1b). Similarly, wild-type Tau seeds (inducer) were less efficient compared to mutated TauP301L seeds, in inducing Tau-aggregation (Fig. S1b, c). Hence, in this work, we analyzed the functional repercussions of Tau-aggregation in optimal conditions, using Tau mutated at Proline 301 acting both as inducer and recipient.

**Fig. 1:**
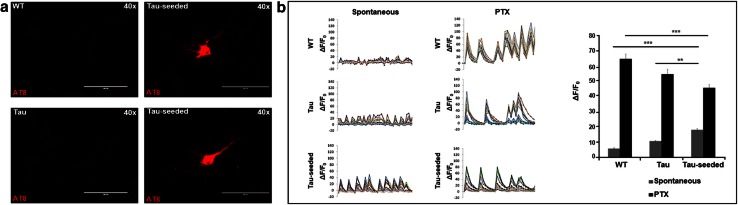
Tau-seed induced Tau aggregation affected neuronal network activity in primary neurons. **a** Tau-seeding in primary neurons from TauP301S mice (Tau-seeded) induced somatic accumulation of aggregated Tau, as detected by AT8 staining following stringent extraction to eliminate soluble Tau, in ~5–10 % of the neurons. AT8 staining was not detected in non-seeded TauP301S neurons (Tau) or neurons derived from non-transgenic mice (WT) (*scale bar* 100 µm). **b** Calcium oscillations in primary neurons were visualized using Fura-2AM [expressed as background corrected increase in fluorescence divided by the resting fluorescence (Δ*F*/*F*_0_)] for the indicated conditions. Representative traces of spontaneously oscillating neurons for each condition (WT neurons, Tau-expressing neurons, Tau-seeded Tau-expressing neurons), reveal higher amplitudes, higher synchronicity, and more regularly shaped oscillations in Tau-seeded neurons, compared to Tau and to WT neurons. Application of picrotoxin (PTX) increased amplitudes of oscillations, the synchronicity and the shape of the oscillations. Mean amplitudes were significantly lower in Tau-seeded neurons following PTX administration compared to Tau and WT neurons. Quantitative analysis of amplitudes in the presence and absence of PTX are presented for the different conditions [number of neurons measured per condition (*n* = 47 (WT); *n* = 66 (Tau); *n* = 68 (Tau-seeded); experiments (*n* = 3); mean ± SEM are presented; **p value <0.01, ***p value <0.001]

To analyze the effect of Tau-seed induced Tau-aggregation on neuronal network function, we performed Tau-seeding in primary neurons derived from TauP301S transgenic mice [27, 63, 73]. Addition of Tau-seeds—without BioPORTER—induced Tau-aggregation in primary Tau-expressing neurons 10 days post-seeding. Tau-aggregation was demonstrated using AT8 staining following stringent extraction, demonstrating strong somatic AT8 staining in Tau-expressing cultures following Tau-seeding. No AT8 positive neurons were detected in non-seeded Tau-expressing neurons or seeded and non-seeded wild type neurons (Fig. 1a, Fig. S2). To assess if Tau-seeded Tau-aggregation affected neuronal network activity, we measured synchronized calcium oscillations using Fura-2 AM dye [44, 55]. Spontaneous calcium oscillations were rather limited in primary wild-type neurons, while amplitudes increased in TauP301S expressing primary neurons, and were significantly more pronounced and well-synchronized in Tau-seeded TauP301S expressing neurons [control (5.50 ± 0.67); Tau-neurons (10.49 ± 0.39); Tau-seeded Tau-neurons (17.89 ± 0.78)] (Fig. 1b). Addition of picrotoxin (PTX)—a non-competitive GABA_A_R-antagonist—known to induce seizures similar as PTZ—in wild-type neurons, increased robustly the amplitude of the oscillations [no PTX (5.50 ± 0.67), with PTX (64.59 ± 3.24)], increased the number of synchronously oscillating neurons and induced regularly shaped responses (characterized by a steep rise and slower decay), as reported previously [44] (Fig. 1b). Of note, similar but less pronounced changes as PTX application are observed when comparing Tau-seeded with non-seeded Tau-expressing and wild-type neurons in unstimulated conditions. This suggests that Tau-seed induced Tau-aggregation acts similar as a GABA_A_R-antagonist, resulting in impaired GABA-ergic transmission. We further identified PTX-induced calcium oscillations as excitatory AMPA- and NMDA-dependent oscillations as they were completely blocked following application of CNQX and D-APV (Fig. S2b), as shown previously [44]. Intriguingly, amplitudes of PTX-induced calcium oscillations were significantly lower in Tau-seeded Tau-expressing neurons (45.53 ± 2.18) compared to non-seeded Tau-expressing (54.51 ± 3.25) or to wild-type neurons (64.59 ± 3.24), indicating that also excitatory transmission was impaired following Tau-seeding (Fig. 1b). Importantly, Tau-seeding in WT—non-transgenic—neurons did not induce Tau-aggregation as analyzed by AT8 staining, and did not affect spontaneous nor PTX-induced calcium oscillations (Fig. S2a, b). Taken together, analysis of spontaneous and PTX-induced neuronal network activity, demonstrated significantly different responses in control, Tau-expressing and Tau-seeded Tau-expressing neurons, suggesting impaired GABA-ergic and excitatory synaptic transmission following Tau-seeding. Hence, Tau-seeding affects neuronal and neuronal network function. It must be noted that only 5–10 % of Tau-seeded neurons displayed strong somatic AT8 staining. Thus, either a few neurons can have a robust impact on neuronal network activity, or alternatively and most parsimoniously, other pathological forms of Tau contribute to impaired neuronal network function.

### Synthetic Tau seed induced Tau-aggregation impaired basal synaptic transmission and synaptic plasticity in organotypic hippocampal slices

**Fig. 2:**
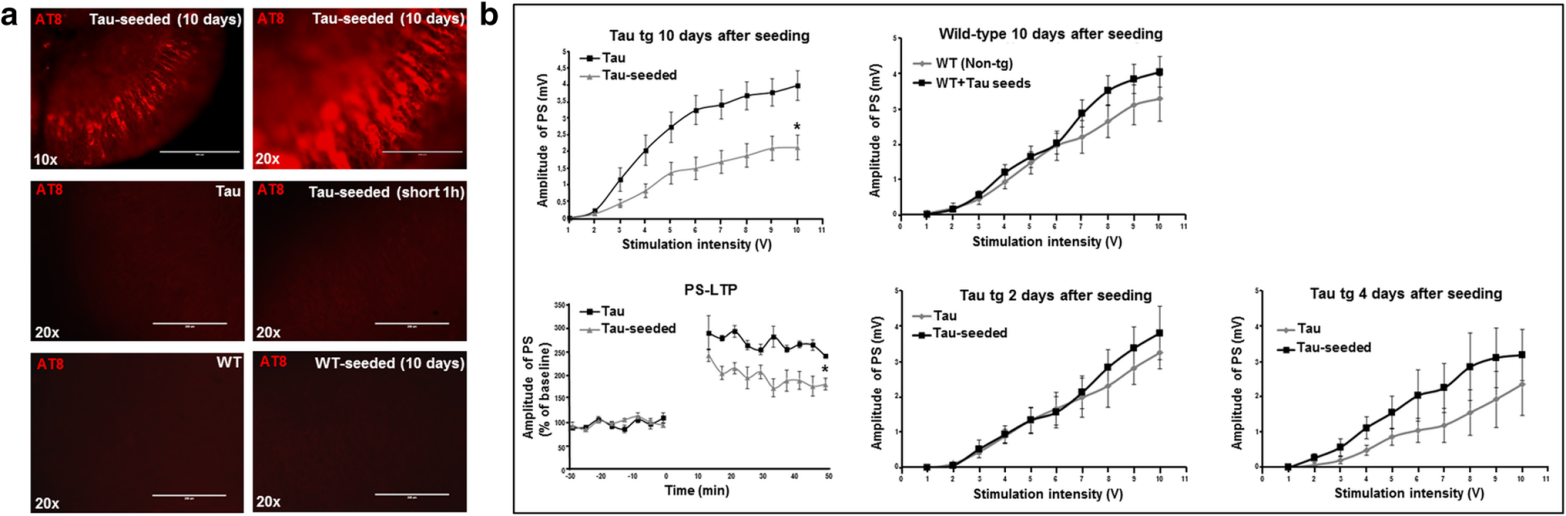
Synthetic Tau seed induced Tau-aggregation impaired basal synaptic transmission and synaptic plasticity in organotypic hippocampal slices. **a** Tau-aggregation induced by Tau-seeding at DIV3 and DIV6 in organotypic hippocampal slices derived from P6 heterozygous TauP301S transgenic pups. AT8 staining revealed robust AT8 staining in hippocampal CA1 region 10 days after seeding of TauP301S organotypic cultures [Tau-seeded (10 days)], not detected in neurons of non-seeded TauP301S organotypic cultures (Tau), short application (1 h) of Tau-seeds [Tau-seeded (short (1 h)] on TauP301S organotypic cultures (OC) or organotypic cultures derived from non-transgenic mice (WT) with or without Tau-seeding (10 days) (*lower panel*) (*scale bar* ×10—400 µm, ×20—200 µm). **b** Population spike (PS) measurement in CA1 following stimulation of Schaffer collaterals. Amplitudes of population spikes were significantly decreased 10 days after seeding in Tau-seeded compared to non-seeded TauP301S cultures [*n* = 15 (Tau); *n* = 17 (Tau-seeded); mean ± SEM are presented; *p value <0.05]. LTP of the population spike amplitude measured following tetanic stimulation of Schaffer collaterals was significantly impaired 10 days after seeding in Tau-seeded compared to non-seeded slices [*n* = 3 (Tau); *n* = 5 (Tau-seeded); mean ± SEM are presented; *p* value <0.05]. Amplitudes of population spikes were not significantly altered at 2 days [Tau (*n* = 6), Tau-seeded (*n* = 9)] or 4 days [Tau (*n* = 11), Tau-seeded (*n* = 9)] after Tau-seeding in TauP301S cultures or in non-transgenic (wild-type, WT) cultures 10 days after Tau-seeding [WT (*n* = 10); WT + Tau-seeds (*n* = 7)]

We next extended our assay ex vivo, in organotypic hippocampal cultures derived from 6 days old Tau P301S transgenic pups [64, 73]. Consecutive addition of Tau-seeds at DIV3 and DIV6 induced robust Tau-aggregation in hippocampal CA1 neurons 10 days after the first seeding, demonstrated by AT8 staining in hippocampal slices while absent in non-seeded hippocampal Tau slices or in seeded and non-seeded hippocampal slices derived from non-transgenic mice (Fig. 2a). Basal synaptic excitability as well as synaptic plasticity were measured in hippocampal CA1 region following stimulation of Schaffer collaterals. This revealed impaired population spikes, after 10 days of seed induced Tau aggregation in Tau hippocampal slices. No significant changes in basal synaptic excitability were detected in wild-type hippocampal slices 10 days after Tau-seeding, nor in Tau-expressing hippocampal slices at 2 or 4 days after seeding (Fig. 2b). This indicates that sufficient induction of Tau-aggregation is required to induce synaptic dysfunction. Furthermore, long-term potentiation of population spikes amplitude induced by tetanic stimulation of Schaffer collaterals revealed impaired synaptic plasticity in Tau-seeded compared to non-seeded Tau hippocampal slices (Fig. 2b). Our data indicate significantly impaired synaptic plasticity and basal synaptic function by Tau-seed induced Tau-aggregation.

### Tau aggregation following seeding with synthetic pre-aggregated Tau spreads to synaptically and functionally connected brain regions in TauP301S transgenic mice

**Fig. 3:**
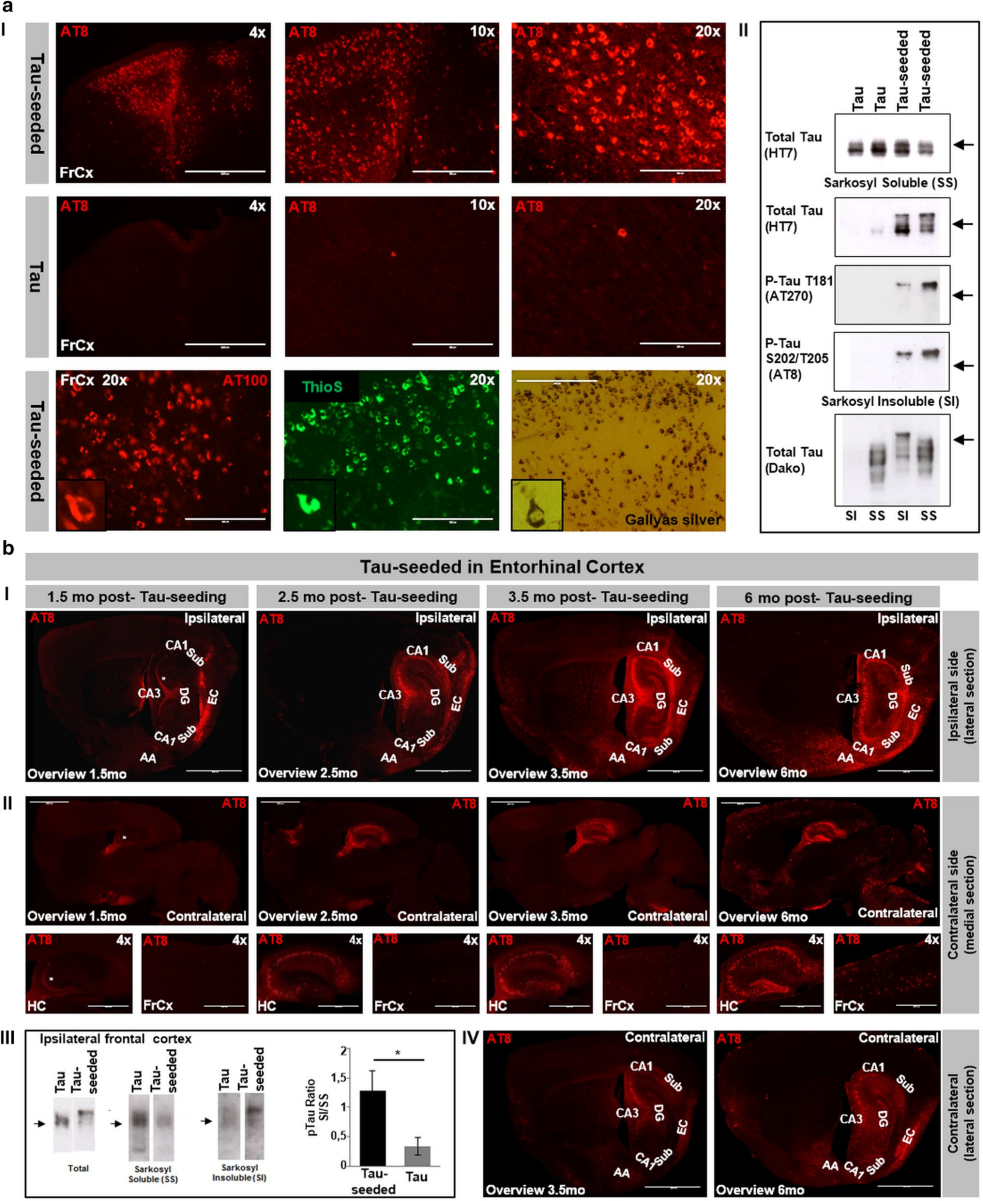
In vivo aggregation of Tau following injection of Tau-seeds. **a** Tau-seeding induced Tau-aggregation in vivo. *I* Intracerebral injection of Tau-seeds in frontal cortex and hippocampus (not shown) at 3 months in TauP301S transgenic mice induced robust Tau-pathology 6 months post-injection, in contrast to buffer only injected TauP301S mice or non-injected TauP301S mice (not shown). Representative AT8 stainings are shown (magnification ×4, ×10, ×20). Staining with AT100, Gallyas silver and ThioS are presented (magnification ×20) (*scale bar* ×4—1000 µm, ×10—400 µm, ×20—200 µm). *II* Biochemical analysis revealing Tau-seed induced Tau-aggregation, as demonstrated by immunoblotting of sarkosyl soluble and sarkosyl insoluble fractions of brain extracts. Total levels of sarkosyl soluble Tau were not significantly affected following Tau seeding, but show increased ratio of higher MW to lower MW Tau forms. Marked increase in sarkosyl insoluble Tau following Tau-seeding as revealed by immunoblotting with HT7 (total Tau) antibody, AT8 (P-S202/T205) antibody and with AT270 (P-T181) antibody of sarkosyl insoluble fractions extracted from brains of Tau-seeded mice, 6 months post-injection, compared to non-seeded Tau mice. Sarkosyl insoluble Tau displayed a characteristic shift in mobility compared to sarkosyl insoluble Tau (*arrows* indicate 68 kDa). **b** Progressive spreading of Tau-pathology. Longitudinal analysis at different post-injection (p.i.) times (1.5 mo, 2.5 mo, 3.5 mo and 6 mo post-injection). Tau-pathology revealed by AT8 staining spreads progressively from entorhinal cortex (EC) to functionally connected hippocampal regions (CA1, CA3, DG, Sub) and amygdala (AA). At 1.5 mo p.i. NFTs are mainly restricted to EC. Note that weak axonal staining (indicated with *asterisk*) of mossy fibers and in DG (at 1.5 mo p.i.), does not represent NFTs but axonal staining observed in young TauP301S mice [73]. *I* Tau-pathology progressively spreads to the contralateral hemisphere. Medial and lateral sagittal sections (cfr Fig. 4a for schematic presentation) of the contralateral hemisphere at different times p.i. are presented (*II*, *IV*), demonstrating spreading of Tau-pathology to distant brain regions along neuronal connections (*scale bar* overviews—2000 µm, ×4—1000 µm). *III* Biochemical analysis of spreading of Tau-pathology to ipsilateral frontal cortex. Western blotting of total homogenate (frontal cortex) revealed a characteristic mobility shift (AT8 antibody) and sarkosyl insoluble Tau in frontal cortex extracts following Tau seeding in EC while absent in non-seeded Tau mice (AD-2 antibody). Quantitative analysis revealed significant increased ratio of sarkosyl insoluble to sarkosyl soluble Tau in frontal cortex following Tau-seeding in EC (*n* = 3 per condition; mean ± SEM are presented; *p < 0.05)

To extend our assay in vivo, Tau-seeds were injected in frontal cortex and hippocampus of TauP301S mice and analyzed for Tau-aggregation in vivo. TauP301S transgenic mice develop Tau hyperphosphorylation, robust Tau-pathology, astro- and microgliosis, hippocampal atrophy, subtle defects in synaptic plasticity and motoric deficits, followed by the development of a progressive neurodegenerative phenotype characterized by initial clasping, hindlimb paralysis, development of hunchback, followed by premature death [73]. In the colony bred in our lab, this phenotype starts at around 10–11 months [35, 63]. Hence, TauP301S mice were used for the Tau-seeding assay at the age of 3 months and analyzed 6 months post-injection—or earlier—before robust Tau-pathology or phenotypic changes were detected in non-seeded TauP301S mice. After a single injection of Tau-seeds in frontal cortex and hippocampus, robust Tau-pathology was demonstrated while practically absent in control injected, non-seeded TauP301S mice (Fig. 3aI). Biochemical analysis demonstrated the presence of sarkosyl insoluble Tau-aggregates following Tau-seeding (Fig. 3aII). Pathological Tau-alterations—including the characteristic 68 kDa Tau—were revealed by immunoblotting of sarkosyl insoluble and soluble fractions with anti-total Tau (HT7) antibody, anti-Tau P-S202/T205 (AT8) antibody, anti-Tau P-T181 (AT270) antibody and anti-Tau P-T212/TS214 (AT100) antibody (not shown) and by the characteristic mobility shift between sarkosyl soluble and sarkosyl insoluble Tau (Fig. 3aII). The presence of fully mature NFTs, containing fibrillary Tau, was further confirmed by Gallyas silver and ThioS staining (Fig. 3aI).

**Fig. 4:**
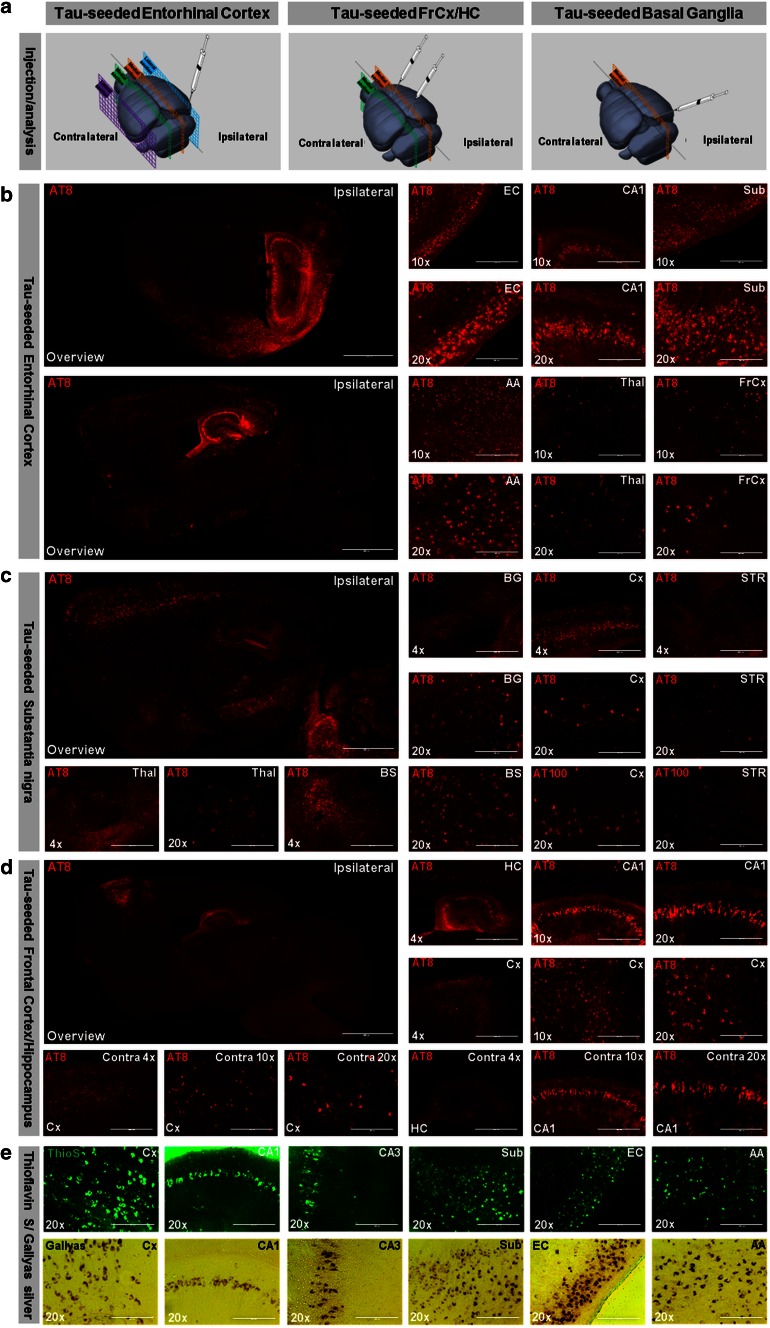
Seeding and spreading of Tau-aggregation according to connectivity rather than proximity and along disease relevant patterns. Seeding and spreading of Tau-pathology following injection of Tau-PFFs in entorhinal cortex (**b**), in substantia nigra (**c**) or in frontal cortex and hippocampus (**d**), injection sites and sections used for analysis are schematically presented in **a** (modified after Allen Mouse Brain Atlas, website: http://mouse.brain-map.org/). Distinct patterns of Tau-pathology are observed. **b** Six months post-injection in entorhinal cortex; AT8 staining is detected in entorhinal cortex (EC), hippocampal Cornu Ammonis (CA1), subiculum (Sub), amygdala (AA), thalamus (Thal) and frontal cortex (FrCx) (*right panel*). An overview of AT8 staining of a section closely to the medial longitudinal fissure and a more lateral section are presented (*left panel*). **c** Six months post-seeding in substantia nigra; AT8 staining is detected in substantia nigra (SN), striatum (STR), thalamus (Thal), brain stem (BS) and motor cortex (Cx). AT8 stained neurons were also detected with AT100 antibody. **d** Six months post-injection in frontal cortex and hippocampus, Tau-pathology was observed at the contralateral hemisphere in cortical (Cx) and hippocampal (HC; CA1) regions, but less pronounced (marked as contra;* lower row*). **e** AT8 stainings used throughout this analysis matched with Gallyas silver and ThioS stainings, as demonstrated in different brain regions (Cx; CA1; CA3, Sub; EC; AA) (*scale bar* overviews—2000 µm, ×4—1000 µm, ×10—400 µm, ×20—200 µm)

To mimic the spreading pattern of Tau-pathology as observed in AD, we injected Tau-seeds in entorhinal cortex (EC) (Figs. 3b, 4b). Tau pathology was initially restricted to EC at 1.5 months post-injection, while gradually extending to functionally connected brain regions over time (Fig. 3bI). From 3.5 months post-injection onwards Tau pathology had spread to hippocampus, amygdala and cortical regions remote from the injection site (Fig. 3bI). NFTs were detected in EC, subiculum, hippocampal formation, amygdala, thalamus and frontal cortex (Fig. 3bI). Furthermore, Tau aggregation was also progressively induced in the contralateral hemisphere (Fig. 3bII, IV). Biochemical analysis of Tau-pathology confirmed the presence of aggregated Tau remote from the injection site, following dissection of ipsilateral frontal cortex of TauP301S mice 6 months post-injection in EC (Fig. 3bIII). This was also reflected in the characteristic shift in mobility in SDS PAGE of Tau in total brain extracts and increased ratio of sarkosyl insoluble to sarkosyl soluble Tau, with a pronounced presence of the 68 kDa band in the sarkosyl insoluble Tau fraction compared to non-injected mice. Noteworthy, injection of Tau-seeds in EC of non-transgenic mice did not induce Tau-pathology, detectable by AT8 staining analyzed 3.5 and 12 months post-injection (Fig. S4).

**Fig. 5:**
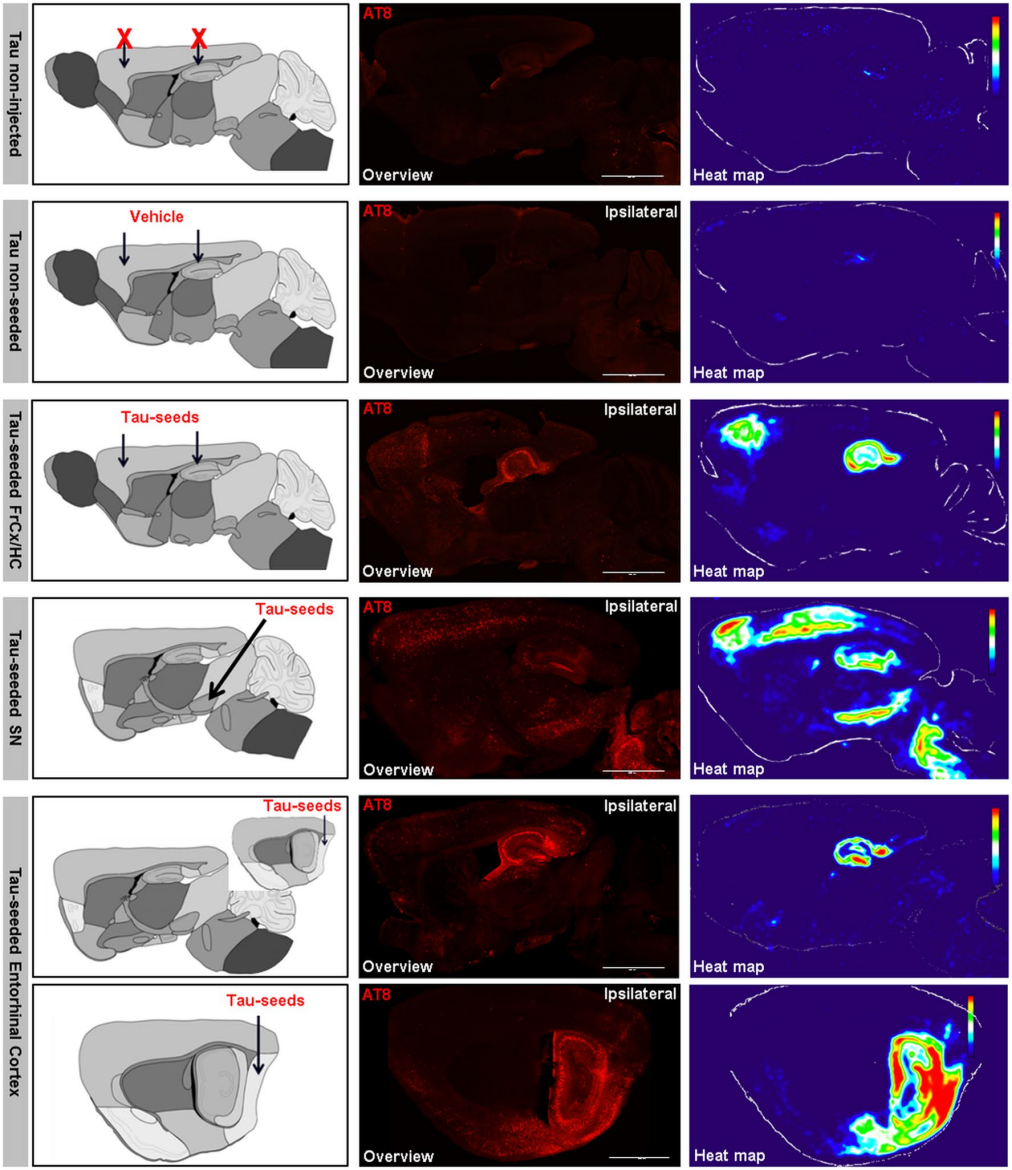
Averaged heat maps for semi-quantitative analysis of spreading of Tau-pathology according to connectivity rather than proximity and along disease relevant patterns. Heat maps representing *color* scored averaged images of AT8 staining were generated for different injection paradigms. Heat maps of Tau mice, of Tau mice injected with vehicle, Tau mice injected with Tau-seeds in frontal cortex and hippocampus (FrCx/HC), Tau mice injected with Tau-seeds in substantia nigra (SN) and Tau mice injected with Tau-seeds in entorhinal cortex (EC), generated using Image J are presented. For Tau mice injected in EC, 2 different sagittal sections are presented situated closely to the medial longitudinal fissure and lateral. Schematic illustrations of the injection site and the section used for analysis are presented in the *first column* (modified after The GENSAT Project, website: http://www.gensat.org). *Arrows* indicate the injection site. Representative AT8 stainings (*middle column*) used for semi-quantitative analysis are presented for each condition, 6 months post-injection of Tau-seeds (*scale bar* 2000 µm). This reveals distinct patterns of Tau-pathology, encompassing brain regions with densely packed Tau-pathology, brain regions with scarcer Tau-pathology and unaffected brain regions. Heat maps were generated for semi-quantitative analysis of Tau-pathology (*right column*) by averaging AT8 stainings of multiple mice for each injection paradigm using Image J (*n* ≥ 3 for each condition). This demonstrates distinct patterns depending on the injection site, along functionally connected brain regions

We next initiated Tau-pathology in basal ganglia, a brain region involved in different functions including motor control, as reflected in its dysfunction in Parkinson’s disease and Parkinsonism. From 3.5 months onwards and at 6 months post-injection of Tau-seeds in basal ganglia (in substantia nigra) Tau aggregation was detected in striatum, thalamus, brain stem and cortical regions including the motor cortex (Fig. 4c). To assess patterns of spreading of Tau-pathology semi-quantitatively we generated averaged heat maps of AT8-stained Tau-pathology in different mice, 6 months post-injection (*n* ≥ 3 for each condition) (Fig. 5). Our standardized AT8 staining protocol to stain forms of Tau, correlating strongly with Gallyas silver and ThioS staining (Fig. 4e) in brains was used [63]. The respective heat maps demonstrated that the spreading pattern of Tau-pathology at 6 months post-injection is dependent on the site of initiation and occurs along functionally connected brain circuits. However, it must be considered that some regions with a more scattered induction of Tau pathology including striatum and thalamus may be underrepresented in the heat maps. This is due to technical issues, because scattered NFTs are more easily leveled out compared to densely packed neuronal layers in the averaging process (Fig. 5).

Taken together, our results indicate that depending on the initial site of injection of Tau-seeds, i.e. cortical/hippocampal, basal ganglia or entorhinal cortex (Fig. 4a–d), different spreading patterns of Tau-pathology to functionally—and often symptomatically—connected brain regions was observed (Fig. 5). Spreading of Tau-pathology occurred by connectivity rather than proximity, raising the question and allowing us to analyze whether neuronal network function is affected by this process.

### Tau-pathology induced by Tau-seeding impairs synaptic function in hippocampal CA1 region and cognition in TauP301S mice

**Fig. 6:**
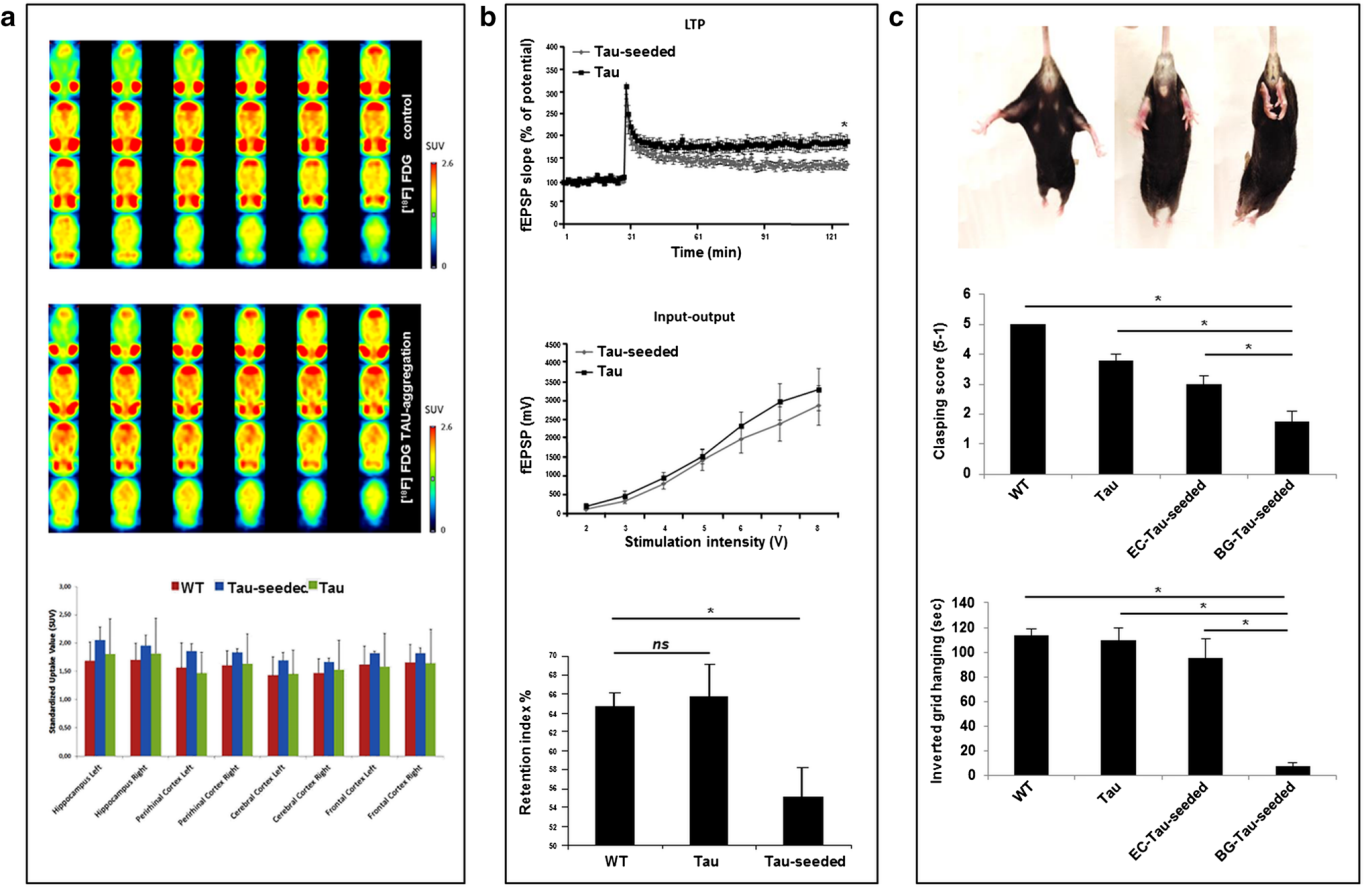
Functional repercussions of Tau seed induced Tau-aggregation along unique neuronal networks, analyzed at 6 months post-injection. **a** FDG-PET analysis: mean horizontal small-animal FDG-PET images of the brain showing two representative samples of Tau-seeded and non-seeded Tau mice. *Color bars* indicate standardized uptake values for [^18^F]FDG. Quantitative analysis of the standardized uptake value revealed no significant difference between the different analyzed groups [non-transgenic (WT), non-seeded Tau transgenic (Tau), Tau-seeded Tau transgenic mice (Tau-seeded); *n* = 3 per group]. **b** Tau-seeded Tau-aggregation causes synaptic and cognitive dysfunction. Electrophysiological analysis of Tau transgenic mice seeded in entorhinal cortex or non-seeded analyzed 6 months post-injection reveals no significantly altered basic synaptic transmission in hippocampal CA1 region [Tau-seeded (*n* = 12); Tau (*n* = 12)]. Long term potentiation (LTP) induced by tetanic stimulation of Schaffer collaterals was significantly impaired following seeding in EC, 6 months post-injection [Tau-seeded (*n* = 5); Tau (*n* = 6); mean ± SEM are presented; *p* value <0.05; Student’s *t* test]. Cognitive function measured by the object recognition task (**b**, *lower panel*). Tau-transgenic mice with bilateral injection of Tau-seeds displayed significantly impaired object recognition memory compared to non-seeded Tau-transgenic mice or non-transgenic WT mice, while Tau transgenic mice were not significantly different compared to non-transgenic WT mice [*n* = 10 (control), *n* = 8 (non-seeded Tau transgenic mice), *n* = 8 (Tau-seeded Tau transgenic mice); mean ± SEM are presented; *p value <0.05; single ANOVA, post-Dunnett’s test]. **c** Motor impairment following initiation of Tau-seeding in basal ganglia, but not in entorhinal cortex. Representative images of clasping scores are presented (*upper panel*) from left to right: score 5 (no clasping); score 3 (intermediate clasping); score 1 (severe clasping). Clasping and inverted grid hanging was assessed for wild type mice (WT; *n* = 12), non-injected Tau mice (Tau; *n* = 6), Tau mice following Tau-seeding in entorhinal cortex (EC-Tau-seeded; *n* = 5), and Tau-mice following seeding in basal ganglia (BG-Tau-seeded; *n* = 7) 6 months post-seeding. Quantitative analysis revealed significantly increased clasping and impaired inverted grid hanging in Tau mice following seeding in basal ganglia, compared to Tau mice seeded in EC, Tau mice and non-transgenic mice (mean ± SEM are presented; *p value <0.05; one-way ANOVA, post-Dunnett’s test). In the graph in panel **c**, non-injected Tau-mice are presented for comparison with Tau-seeds injections in EC and basal ganglia. Sham injections in Tau mice in basal ganglia [clasping score: 3.9 ± 0.4, inverted grid hanging: 98.9 ± 12.7 (mean ± SEM, *n* = 9)] did not alter clasping or inverted grid hanging scores compared to non-injected Tau mice [clasping score: 3.8 ± 0.2, inverted grid hanging: 109.4 ± 10.0 (mean ± SEM, *n* = 6)] (not presented in the graph)

Disease-specific patterns of glucose metabolism are generally considered to reflect neuronal dysfunction and have been demonstrated to correlate with high NFT load in different Tauopathies. To analyze the functional repercussions of spreading of Tau-aggregation through neuronal networks dependent on the site of initiation, we used in first instance FDG-PET. Mice injected with Tau-seeds and control mice were subjected to small-animal FDG-PET at 6 months post-injection. However, no significant differences were detected between Tau-seeded and non-seeded mice, despite the presence of robust Tau-pathology (Fig. 6a). This lack of changes should be ascribed to a lack of neurodegenerative processes induced by Tau-seeded Tau-aggregation, to the fact that neuronal dysfunction precedes significant changes in FDG-PET, or to the fact that changes in FDG-PET measured glucose metabolism were too small to detect with the current technique. Although, this technique was used successfully to reveal differences in glucose metabolism following contextual fear conditioning in rodents, and in different AD models [43, 46]. To gain further insight, we analyzed synaptic and cognitive function following Tau-seed induced Tau-aggregation. We assessed whether induction of Tau-aggregation through functional connections by Tau-seeding in EC, affected synaptic function in hippocampal neurons of CA1 following stimulation of the Schaffer collaterals 6 months post-seeding. Analysis of synaptic plasticity demonstrated a defect in LTP following Tau-aggregation in hippocampal CA1 neurons after induction of Tau-seeding in EC, while basal synaptic transmission was not affected in hippocampal CA1 neurons (Fig. 6b). Synaptic fatigue and paired pulse ratio—generally considered parameters of presynaptic function—were not affected by Tau-seeding, suggesting a postsynaptic rather than presynaptic deficit (Fig. S3). In line with this, immunohistological staining with the presynaptic marker SV2 revealed no alterations following Tau-seeding in entorhinal cortex (results not shown). Long-term potentiation is generally believed to present a cellular model of learning and memory formation, and is induced during learning in CA1 [71]. Therefore, we assessed hippocampus-dependent cognitive function following Tau-seed induced Tau-aggregation. We first measured cognitive function after unilateral injection of Tau-seeds in Tau-transgenic mice, revealing no significant changes between unilateral Tau-seeded mice and non-seeded mice (results not shown). We next assessed cognitive function, 6 months following bilateral injections. This revealed a significant deficit in object recognition memory 1 h post-training in Tau-seeded mice compared to non-seeded mice (Fig. 6b). Our data thereby demonstrate that Tau-seeded Tau-aggregation is associated with impaired cognition in Tau-transgenic mice.

### Spreading of Tau aggregation along neuronal circuitries and impairment of associated behavioral outcomes is determined by the site of initial seeding in TauP301S mice

We next analyzed whether the site of initiation of Tau-aggregation determines the specific behavioral outcomes. We measured motoric performance 6 months after initiation of Tau pathology in basal ganglia and EC, respectively. Quantitative scoring of hindlimb clasping revealed significantly increased clasping following initiation of Tau-pathology in basal ganglia, compared to initiation of Tau-pathology in EC, which did not induce significant clasping (Fig. 6c). Furthermore, we measured inverted grid hanging performance, to assess motor coordination and grip strength. Performance in inverted grid hanging was severely impaired 6 months post-initiation of Tau-aggregation by Tau-seeding in basal ganglia, while not significantly affected 6 months post-seeding in EC (Fig. 6c). Intracerebral injections of buffer only in basal ganglia did not induce Tau-aggregation and did not affect clasping or inverted grid hanging (Fig. 6c). Taken together, our data indicate that prion-like seeding of Tau-aggregation along synaptic connections affects selective functional brain circuitries, thereby determining the behavioral dysfunction depending on the site of initiation of Tau-pathology.

### “Early” pathological forms of Tau rather than fully mature NFTs as causal culprits for neuronal dysfunction

**Fig. 7:**
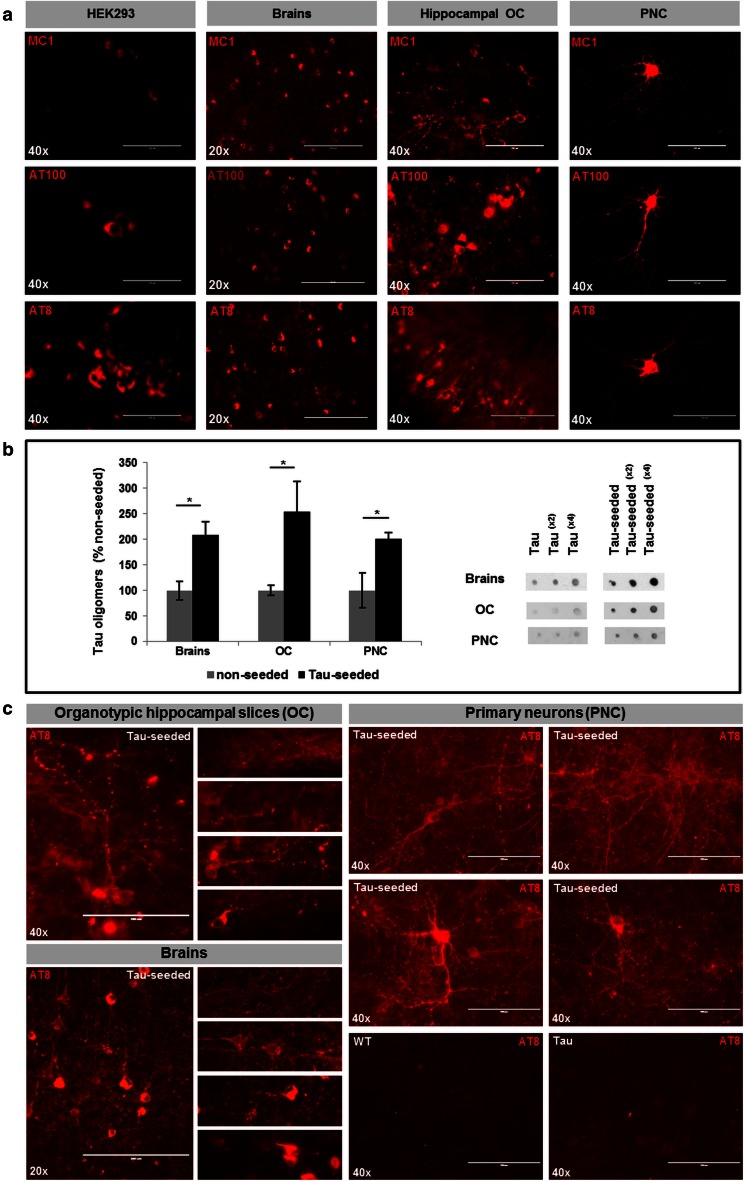
Different pathological forms of Tau are generated by Tau-seed induced Tau-aggregation. **a** Immunostaining of pathological forms of Tau reveals the presence of hyperphosphorylated Tau forms phosphorylated at Ser202/Thr205 (AT8) and T212/S214 (AT100), as well as misfolded Tau (MC1) antibody in all models, i.e. HEK293, brains, organotypic cultures and primary neurons, following Tau-seeding (*scale bar* ×20—200 µm, ×40—100 µm). **b** Tau-seeding increased Tau-oligomer concentrations assessed by dot blotting analysis with the Tau-oligomer specific T22 antibody on total homogenates. Quantitative analysis demonstrated accumulation of oligomeric Tau forms following Tau-seeding in brains [(*n* = 4; *n* = 4), (non-seeded; Tau-seeded)], organotypic cultures (OC) (*n* = 5; *n* = 5) and primary neurons (PNC) (*n* = 6; *n* = 5) (mean ± SEM are presented; *p value < 0.05). Dilution curves of samples of the different models are presented. **c** Immunohisto/cytological analysis with AT8 reveals different forms of AT8 stained Tau. In addition to mature and immature NFTs, diffuse and punctated staining are prominently observed by AT8-staining in organotypic hippocampal cultures and brains following Tau-seeding (*left panels* provide an overview picture and details of different types of AT8 staining observed in Tau-seeded OC and brains). In PNC, AT8 staining following standard fixation in 4 % PAF, demonstrates strong diffuse and punctated AT8 staining present throughout the culture dish in PNC, and robustly induced following Tau-seeding in TauP301S expressing PNC. *Lower panels* show AT8 staining (standard fixation) in non-seeded primary neurons derived from TauP301S mice (Tau) or non-transgenic mice (WT) (scale bar ×20–200 µm, ×40–100 µm)

To gain insight in potential forms of Tau responsible for the neuronal network dysfunction observed in our models, we performed biochemical and immunological analyses. We first assessed the induction of pathological Tau-forms in brains in vivo following Tau-seeding. Immunohistological analysis demonstrated the presence of misfolded Tau (MC1) and Tau hyperphosphorylated at pathological epitopes S202/T205 (AT8) and T212/S214 (AT100) (Fig. 7a). The presence of fully mature NFTs was further confirmed by Gallyas silver staining and ThioS staining (Figs. 3aI, 4e). Finally, electron microscopy (EM) analysis confirmed the clear presence of Tau fibrils and aggregated Tau (Fig. S5). Dot blot analysis with T22 antibody [40], demonstrated accumulation of Tau-oligomers in total homogenates following Tau-seeding (Fig. 7b). Tau-multimers were further analyzed in total homogenates in non-denaturing and non-reducing conditions in native PAGE. Larger Tau-aggregates, i.e. granular Tau (~40 Tau-molecules, 2.4 MDa) and fibrillary Tau, cannot enter the gel under these conditions. AT8 positive Tau-multimers were increased in brains following Tau-seeding (Fig. S6). To gain more insight in early pathological forms of Tau, we performed a more detailed immunohistological analysis using AT8 staining. This demonstrated the presence of clearcut NFTs, which we previously demonstrated to correlate with Silver staining and ThioS staining in brains, but using more sensitive settings also different forms of AT8-stained Tau were revealed. More particularly, neurons with different stages of Tau-pathology ranging from early to fully mature NFTs were observed. In addition, diffuse and punctate staining patterns were observed in broader regions than regions displaying mature NFTs (Fig. 7c). This diffuse and punctate staining was markedly induced by Tau-seeding (Fig. S7) and observed in cell bodies but also very prominently in neuritic extensions of neurons. Taken together, Tau-seeding propagated diverse pathological Tau forms ranging from clearcut mature NFTs (somatic fibrillar Tau-aggregates) to “early” pathological forms of Tau, encompassing more diffuse and punctate AT8, as well as hyperphosphorylated, misfolded (MC1) and oligomeric Tau forms (stained with T22 or AT8).

We next analyzed the presence of these different forms of Tau following Tau-seed induced Tau-aggregation in primary neurons (PNC) and organotypic cultures (OC) (Fig. 7). Tau-seeding induced more robust and more abundant AT8 staining in organotypic hippocampal slices than in primary neurons, due to higher concentrations of Tau-seeds and topical application of the seeds on the slice. In primary neurons Tau-seed induced Tau-aggregation was less robust than previously published [27] since lower concentrations of Tau-seeds were used. Immunological analysis revealed the presence of pathological forms of Tau reflected by AT8, AT100 and MC1 positive signals in both models, OC and PNC (Fig. 7a). EM analysis revealed the presence of Tau fibrils in OC following Tau seeding, albeit less abundant than in brains (Fig. S5). Fibrils were not detected by EM analysis in primary neurons, paralleled by the lack of ThioS staining in PNC (results not shown). This indicates that fully mature Tau-fibrils were absent or present in very low quantities below detection limit of the assay (EM and ThioS) in PNC. Oligomeric Tau forms were increased in primary neurons and organotypic cultures as demonstrated by dot blotting with the Tau oligomer-specific T22 antibody (Fig. 7b). In both models, Tau-seeding increased AT8-positive Tau-multimers detected in Native PAGE (Fig. S6). To further analyze early pathological forms of Tau in PNC, we performed a more sensitive AT8 staining, by omitting the stringent extraction of soluble forms of Tau, thereby staining both aggregated and soluble forms of AT8-stained Tau. This revealed marked Tau-seed induced diffuse and punctate AT8-staining, in addition to the somatic Tau-aggregation as previously detected (Fig. 7c). As indicated above, strong somatic NFT-like Tau-staining of aggregated Tau was only observed in 5–10 % of the neurons in PNC. In contrast, the punctate and more diffuse staining pattern was robustly observed throughout the culture (Fig. 7c), and was markedly induced by Tau-seeding in Tau-expressing neurons and absent in wild-type neurons (Fig. S7). This punctate and diffuse staining pattern was also observed in neurons not yet displaying strong NFT-like somatic AT8 signal, hence representing an early form of pathological AT8 positive Tau. In view of the detection of somatic aggregated Tau in only very limited number of neurons, and the inability to detect fibrillar Tau in primary neurons following Tau-seeding using EM and ThioS, the combined data point to early pathological forms of Tau, encompassing non-NFT AT8-positive Tau and oligomeric Tau, rather than fully mature fibrillary NFTs, as culprit for Tau-seed induced neuronal dysfunction in PNC. The clearcut presence of these early pathological Tau-forms in brains and organotypic hippocampal slices following Tau-seeding, further supports their potential role in neuronal dysfunction in all models. Taken together, our data suggest that early pathological forms, encompassing diffuse and punctate AT8 stained Tau, hyperphosphorylated, misfolded (MC1) and oligomeric Tau forms (stained with T22 or AT8), rather than fully mature NFT, correlate with the neuronal dysfunction induced by Tau-seeded Tau-misfolding.

## Discussion

Accumulating evidence has demonstrated that Tau displays prion-like properties and that prion-like spreading of Tau-pathology occurs through connectivity rather than proximity [1]. This process has been proposed to underlie the stereotyped progression along unique brain circuitries of Tau-pathology and symptoms in Tauopathies [22, 28, 50, 53, 58, 74]. In this work, we demonstrated—for the first time—that templated Tau-misfolding induced by Tau-seeds through functionally connected neuro-anatomical pathways, impairs neuronal network function and associated behavioral outcomes, dependent on the initial site of seeding. More precisely, we demonstrated that small amounts of pre-aggregated synthetic Tau fragments, focally injected, without changing expression-level of Tau but only inducing Tau-misfolding, are sufficient to propagate Tau aggregation and impair neuronal network function and behavioral outcomes 6 months post-injection, dependent on the initial site of injection. These findings emphasize the potential relevance of this intensively studied process in the stereotypic progression of symptoms in Tauopathies. We have further extended our analysis to highlight early pathological forms of Tau, rather than fully mature fibrillar NFTs as causal culprits for the neuronal network dysfunction.

We demonstrated that templated Tau-misfolding by Tau-seeds affected neuronal network activity in primary neuronal cultures. Primary cortical neurons display synchronized calcium oscillations in culture, which were previously shown to be modulated by PTX, a GABA_A_ receptor antagonist [44]. Exposure of cortical neurons to PTX renders calcium transients to exhibit increased amplitude, more regular frequency and higher synchronicity [44]. A refractory period has previously been identified which may play a role in increased synchronization. Our data indicate a significant dysregulation of spontaneous and PTX-induced calcium oscillations, indicating that Tau-seeded Tau-misfolding affects neuronal network activity. Our findings suggest that Tau-seeding impairs GABA-ergic synaptic transmission, based on the similarity of the effect of PTX and Tau-seeds on spontaneous oscillations. As we further demonstrated that PTX elicited calcium transients are NMDA- and AMPA-dependent as shown previously [44], their reduced amplitudes in Tau-seeded neuronal cultures indicate impaired glutamatergic, NMDA- and AMPA-dependent signaling. We further extended our analysis in models with conserved network architecture, i.e. to organotypic hippocampal slices [21]. In organotypic cultures, we demonstrated impaired population spike amplitude measured in hippocampal CA1 region following stimulation of Schaffer collaterals. This basal synaptic transmission is predominantly generated by excitatory glutamatergic (NMDA/AMPA-mediated) neurotransmission, further corroborating data obtained in primary neuronal cultures. Furthermore, measurement of LTP revealed that synaptic plasticity is impaired in organotypic slices by Tau-seed induced Tau misfolding. Notably, NMDA- and AMPA-receptor functions are critically involved in hippocampal LTP in CA1. Our data were further corroborated in vivo. Electrophysiological analysis in acute hippocampal slices revealed that LTP was affected 6 months post-induction of Tau-pathology by Tau-seeding in vivo, although basic synaptic transmission was not. The latter may be explained by the fact that effects of Tau-aggregation can be more easily compensated in vivo than in vitro, or that the aggregation process occurred more rapidly in vitro, or that organotypic cultures are more sensitive than the intact hippocampus in vivo. It must be noted that electrophysiological analysis in acute hippocampal slices was performed in hippocampal CA1 region, while initial Tau-seeding was performed in entorhinal cortex, indicating that neuronal function remote from the initial seeding site was affected. We further demonstrated conserved presynaptic parameters including paired pulse ratio, synaptic fatigue and SV2 staining, pointing towards a postsynaptic mechanism. Together with the results obtained in primary neurons, indicating impaired NMDA/AMPA-dependent neuronal network function following Tau-seeding, our data are in line with a role of pathological Tau forms in postsynaptic dysfunction, including NMDA/AMPA dysfunction as previously shown [8, 60, 70]. We further assessed behavioral effects, and demonstrated that cognition, measured in an object recognition task was impaired in bilaterally injected mice, 6 months post-injection. Together our data indicate that Tau-seed induced misfolding of Tau, leading to Tau-aggregation through connectivity, affects neuronal function, neuronal network activity and behavioral outcome, and hence could contribute to progression of the symptoms in Tauopathies.

Prion like induction of Tau-pathology—a process which raised considerable scientific interest—has been elegantly demonstrated and analyzed in exquisite detail for different aspects [1, 12–14, 19, 20, 22, 26, 28, 32, 35, 37, 40, 53, 72]. The functional repercussions of this process remained however, to be analyzed. This question is important to evaluate its relevance in the pathogenetic process of Tauopathies. In AD, Tau-pathology is known to progress according to stereotypical and predictable patterns reflected in its incorporation in criteria for the neuropathological diagnosis of AD [6, 34]. The first neurons to be affected are in entorhinal cortex (EC) (stage I), the neurons that give rise to the perforant pathway, the major projection connecting cerebral cortex with the hippocampus [23, 33]. Next NFTs spread to the CA1 region of the hippocampus and accumulate in limbic structures such as the subiculum (stage II–III) and the amygdala, thalamus, and claustrum (stage IV). Finally, NFTs spread to isocortical areas (stage V–VI). Symptoms in AD are strongly correlated with this characteristic appearance of NFTs. In this work, we mimic spreading of Tau-pathology following seeding in entorhinal cortex of TauP301S mice, similar as observed in AD. Tau pathology starts first in the entorhinal cortex, subsequently in CA1, the subiculum and CA3-4 and later in the DG, and finally in neocortical areas (stages V and IV), according to the Braak and Braak stages. Noteworthy, minor discrepancy between spreading of Tau-pathology in AD [EC, subsequently CA1/subiculum/CA3-4 and later dentate gyrus (DG)] and functional connectivity (EC, subsequently DG/CA3-4 and then CA1/subiculum) may be ascribed to additional factors determining selective vulnerability or protection in these regions, which appear to be recapitulated in our model, as the AD pattern was mimicked following seeding in EC. It must be noted however that in contrast to AD and sporadic Tauopathies, characterized by aggregation of wild type Tau, we used in the current work recipient Tau and inducer Tau-seeds mutated at Proline 301, as this combination results in the highest Tau-aggregation efficiency. Previous work also indicated that mutation of Proline 301, rather than its substitution by Serine or Leucine, is determining aggregation efficiency [27]. However, wild type Tau acting as recipient, in combination with either wild-type or mutant Tau seeds as inducer has been demonstrated to be capable of prion-like Tau seeding, albeit much less efficient. The potential of wild type Tau to undergo prion-like Tau seeding allows a (cautious) extrapolation of our data in the context of AD or sporadic Tauopathies, characterized by aggregation of wild type Tau. Hence, neuronal network dysfunction and associated behavioral dysfunctions induced by prion-like seeding and spreading of Tau-pathology along functionally connected circuitries could relate to symptomatic progression in AD and related Tauopathies.

Besides in AD patients, spreading of Tau-aggregation according to a characteristic pattern along functionally connected brain circuitries is also observed in argyrophilic grain disease, a different Tauopathy. Other Tauopathies are very heterogeneous between individuals with identical mutations, including for patients with Tau mutated at Proline 301 (P301L) [4]. This heterogeneity hampers the delineation of a specific progression pattern. However, symptoms progressively affect certain behaviors driven by a particular brain circuitry. We here demonstrate for the first time that the initial site of Tau-seeding determines the behavioral outcomes, resulting in different behavioral outcomes depending on the initiation site. Initiation of prion-like seeding in transgenic mice expressing mutant TauP301S, in entorhinal cortex resulted in spreading of Tau-pathology along functionally connected circuits, resulting in impaired LTP in hippocampal CA1 neurons. Cognition measured in an object recognition task was demonstrated to be impaired following Tau-seeded Tau-aggregation. In contrast, prion-like seeding in basal ganglia resulted in spreading to brain regions involved in motor control, thereby resulting in motor impairments, 6 months after Tau-seeding. Motoric impairments were not observed following injection of Tau-seeds in EC. These findings are reminiscent of the heterogeneity of symptoms in TauP301 patients, where the initial site of Tau seeding by environmental, genetic or accidental factors, may determine the symptomatic outcome (cognitive symptoms or Parkinsonism).

The clearcut demonstration of impaired neuronal network function and behavior by prion-like seeding raises questions about the causal culprit, in terms of pathological forms of Tau. The combination of our functional analysis with biochemical and immunological analysis points to early pathological forms of Tau, including pathological hyperphosphorylated and misfolded Tau and Tau oligomers rather than fully mature NFTs as potential pathological culprits. Notably, strong somatic AT8 aggregation was only observed in 5–10 % of neurons following Tau-seeding in primary neurons. Furthermore, the presence of fully mature fibrils was below detection limit using electron microscopy and ThioS staining was not observed in PNC. It must be noted that Tau-seeding in primary neurons in this work was less robust than previously published [27], probably due to lower concentration of Tau-seeds used. We have however, demonstrated the abundant presence of “early” AT8-positive forms of Tau detected as diffuse staining and punctated synaptic staining in the current model. This staining was present throughout the primary neuronal culture and robustly induced following Tau-seeding. Furthermore, oligomeric forms of Tau stained with T22 or with AT8 were increased following Tau-seeding in all models. Although an approximately, twofold increase may seem rather low, the spatio-temporal characteristics (e.g. at the synapse) of this increase may underlay its pronounced effect on neuronal function. Taken together our data indicate that either a few neurons displaying strong somatic AT8 staining are responsible for impaired neuronal network activity, or most parsimoniously, that early forms of aggregated Tau or “pre-tangle stage” pathological forms of Tau, are responsible for neuronal network dysfunction. This is further supported by their abundant induction in brains and in organotypic cultures following Tau-seeding. Taken together our data suggest that early pathological forms of Tau rather than fully mature NFTs may represent the causal culprits. Final proof can only be delivered by selective elimination of different pathological forms of Tau and analysis of the functional repercussions. Our observations fit however with previous observations using elegant approaches to determine the repercussions of Tau-pathology, and more particularly of NFTs on neuronal function in vivo [18, 30, 38, 48, 52, 54, 61, 65, 66, 69]. This demonstrated either no effects of NFTs in neurons on calcium or Arc responses [38, 52]. Furthermore, using inducible expression of Tau or pro-aggregant Tau, reversibility of synaptic deficits despite the continued presence of NFTs was demonstrated, indicating association of synaptic defects with the aggregation process and early pathological Tau forms, rather than with the full-blown NFTs [3, 30, 54, 61, 65, 66, 69]. These data are in line with clear demonstration of detrimental effects of Tau-oligomers on neuronal function and behavior, as demonstrated following acute injection of oligomeric Tau and immunization with anti-Tau oligomeric antibodies [10, 11, 39, 40]. Our findings are furthermore in line with previous reports demonstrating that Tau overexpression or Tau-alterations affect neuronal function or function of connected neurons [29, 31, 42, 47, 48, 56, 57, 63, 73]. It must be noted that in the current work, Tau-expression is not affected, but only misfolding of Tau is induced by templated seeding by focal administration of small amounts of seeds. We demonstrate that this templated Tau-misfolding is sufficient to propagate neuronal network dysfunction and behavioral outcomes, which correlate with the presence of early pathological forms of Tau, including Tau oligomers, rather than with the presence of fully mature Tau fibrils.

In summary, our data indicate for the first time and unequivocally, using in vitro, ex vivo and in vivo approaches that (i) prion-like Tau-seed induced Tau-aggregation with NFT formation—a mechanism under intensive investigation—causes synaptic and neuronal network dysfunction, resulting in behavioral impairments. Our data thereby indicate that prion-like spreading of Tau-pathology may contribute to progression of disease symptoms in Tauopathies by affecting intrinsic functional critical networks. Furthermore, our data indicate unequivocally that (ii) behavioral outcomes following seeding in Tau P301 transgenic mice, are determined by the initial site of Tau seeding. Hence, motoric problems were demonstrated following Tau-seeding in basal ganglia (substantia nigra), while absent following injection in entorhinal cortex, resulting in cognitive defects. These findings are reminiscent of the heterogeneity of clinical symptoms in familial cases with P301 mutations, in which initial seed formation by environmental, genetic or accidental factors, may determine the behavioral outcomes. Finally, (iii) our results support a role of early pathological forms of Tau, including oligomeric Tau rather than fully mature NFTs as pathogenic culprits of prion-like induced spreading of neuronal dysfunction. Our findings provide a basis to further identify the molecular mechanisms involved in Tau-seed induced synaptic dysfunction and provide a model to develop novel therapeutic strategies targeting Tau-seed induced neuronal dysfunction, and to analyze repercussions on neuronal function induced by different Tau-“strains”.

## Electronic supplementary material

Supplementary material 1 (TIFF 7636 kb)

Supplementary material 2 (TIFF 4289 kb)

Supplementary material 3 (TIFF 816 kb)

Supplementary material 4 (TIFF 3810 kb)

Supplementary material 5 (TIFF 23459 kb)

Supplementary material 6 (TIFF 13614 kb)

Supplementary material 7 (TIFF 5042 kb)

Supplementary material 8 (DOCX 58 kb)

Supplementary material 9 (DOCX 22 kb)
