# Supplementary figures and images for: Templated misfolding of Tau by prion-like seeding along neuronal connections impairs neuronal network function and associated behavioral outcomes in Tau transgenic mice
Source: Acta Neuropathol. 2015 Apr 11;129(6):875–94. doi: 10.1007/s00401-015-1413-4 (PMC4436846; doi:10.1007/s00401-015-1413-4)

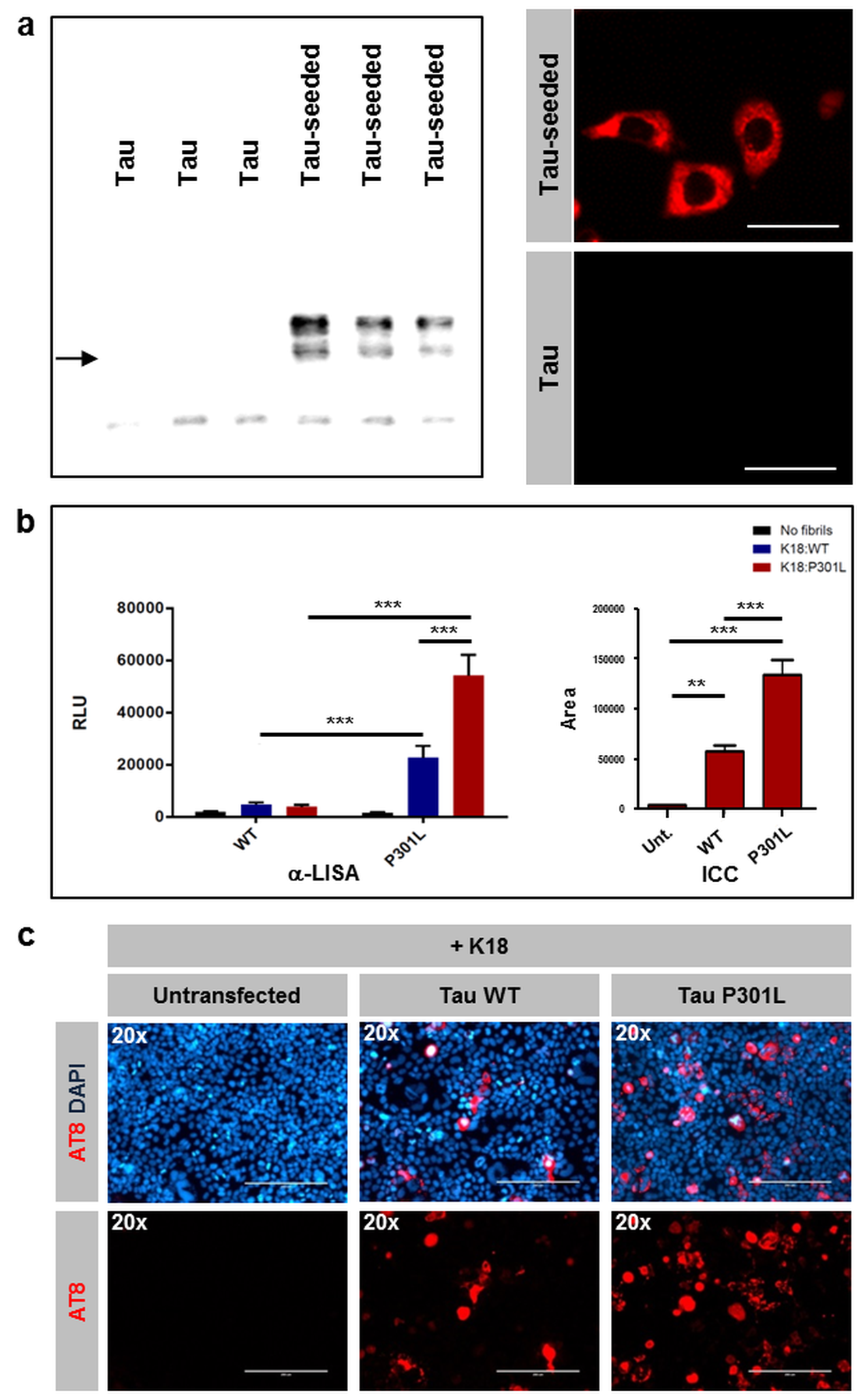

Supplement: Supplementary file 1 — Supplementary material 1 (TIFF 7636 kb) [file 401_2015_1413_MOESM1_ESM.tif]

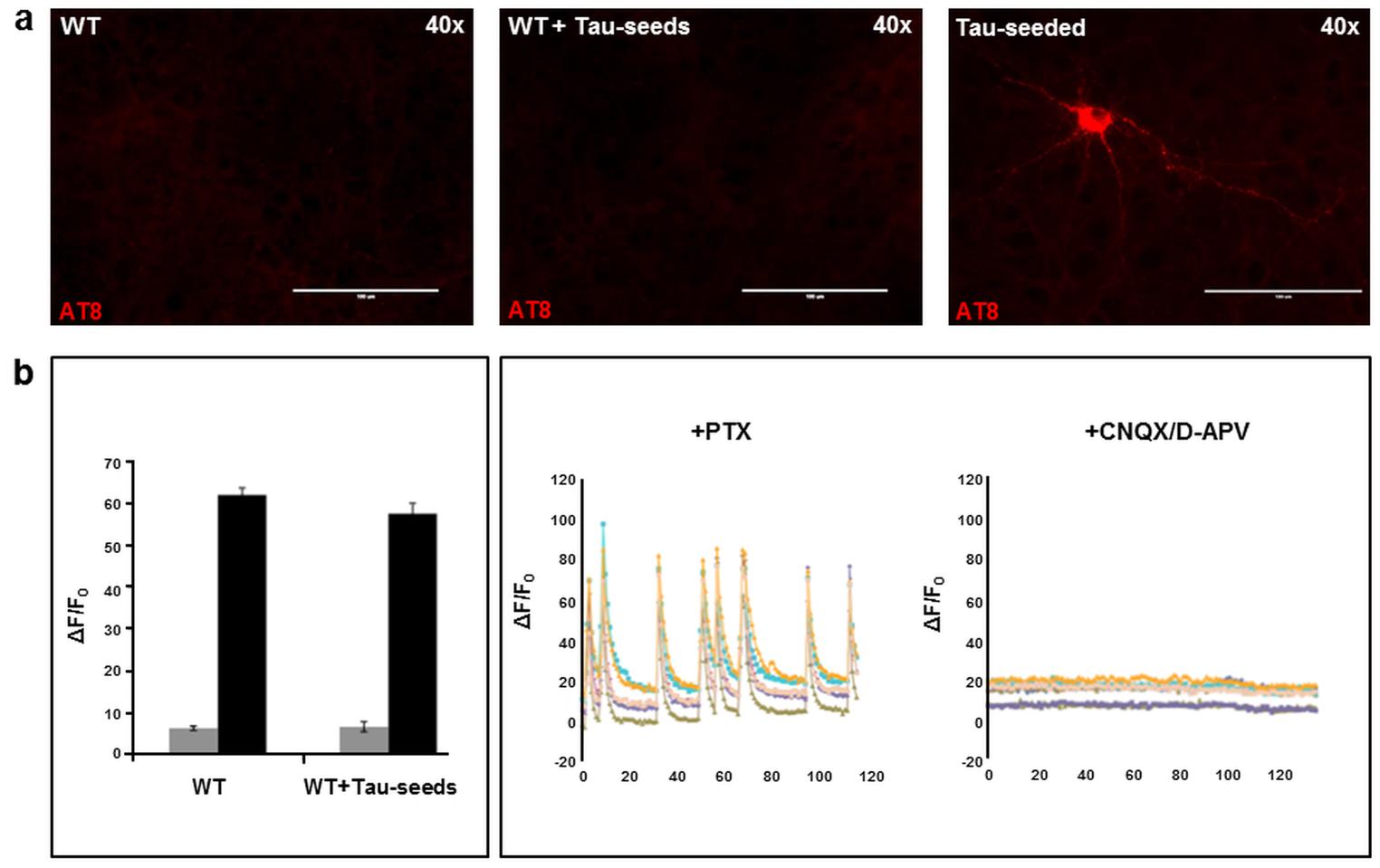

Supplement: Supplementary file 2 — Supplementary material 2 (TIFF 4289 kb) [file 401_2015_1413_MOESM2_ESM.tif]

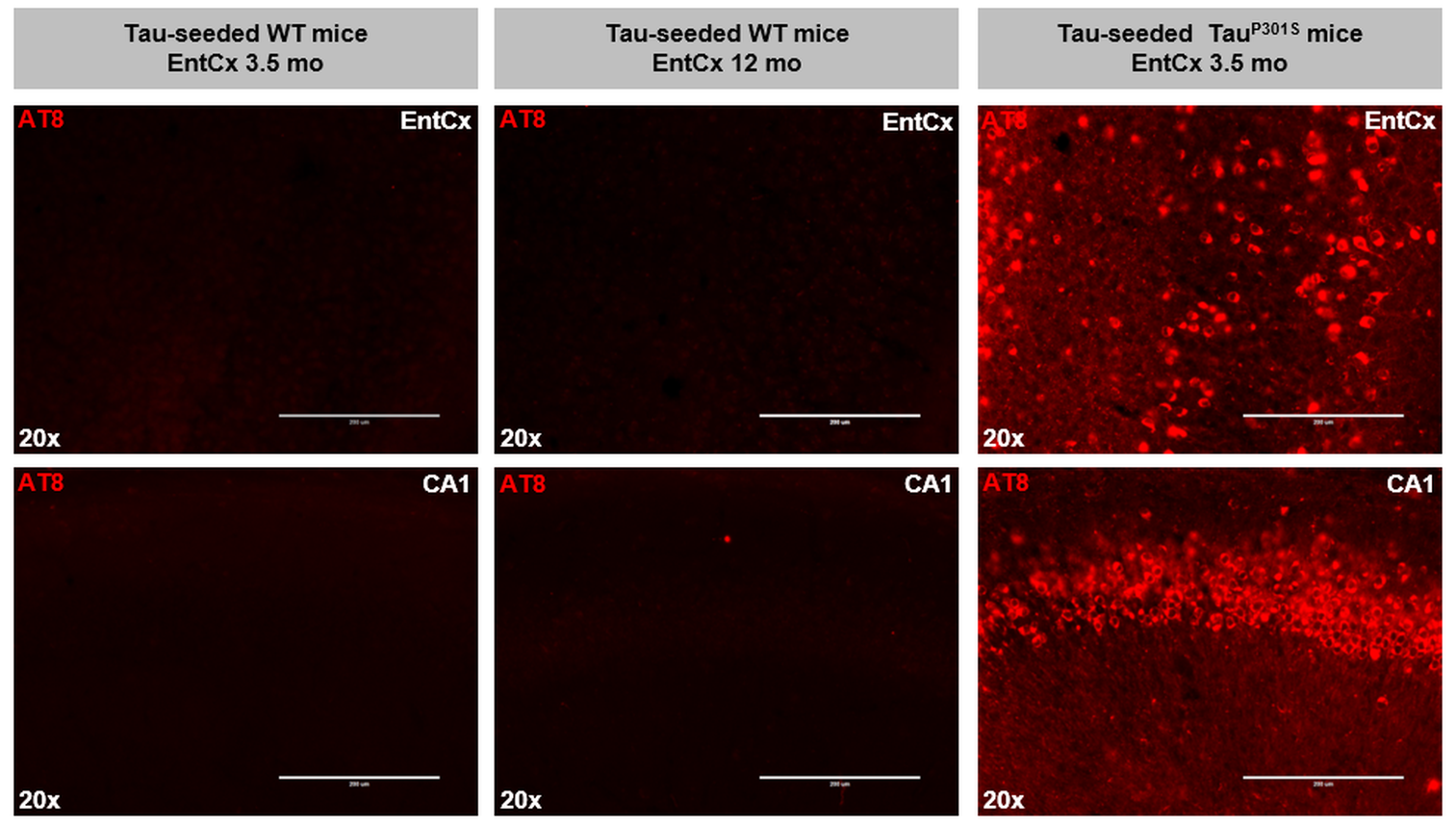

Supplement: Supplementary file 4 — Supplementary material 4 (TIFF 3810 kb) [file 401_2015_1413_MOESM4_ESM.tif]

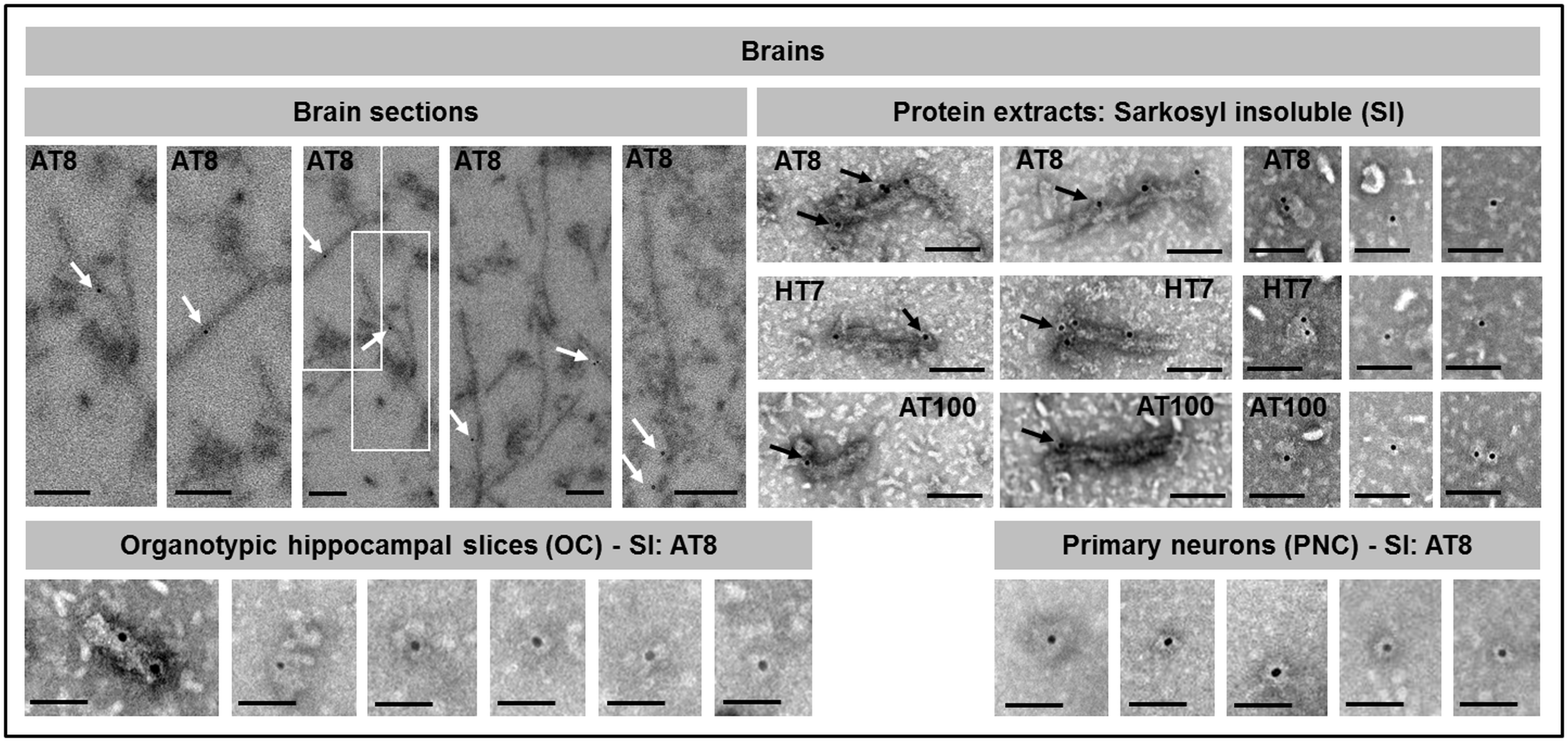

Supplement: Supplementary file 5 — Supplementary material 5 (TIFF 23459 kb) [file 401_2015_1413_MOESM5_ESM.tif]

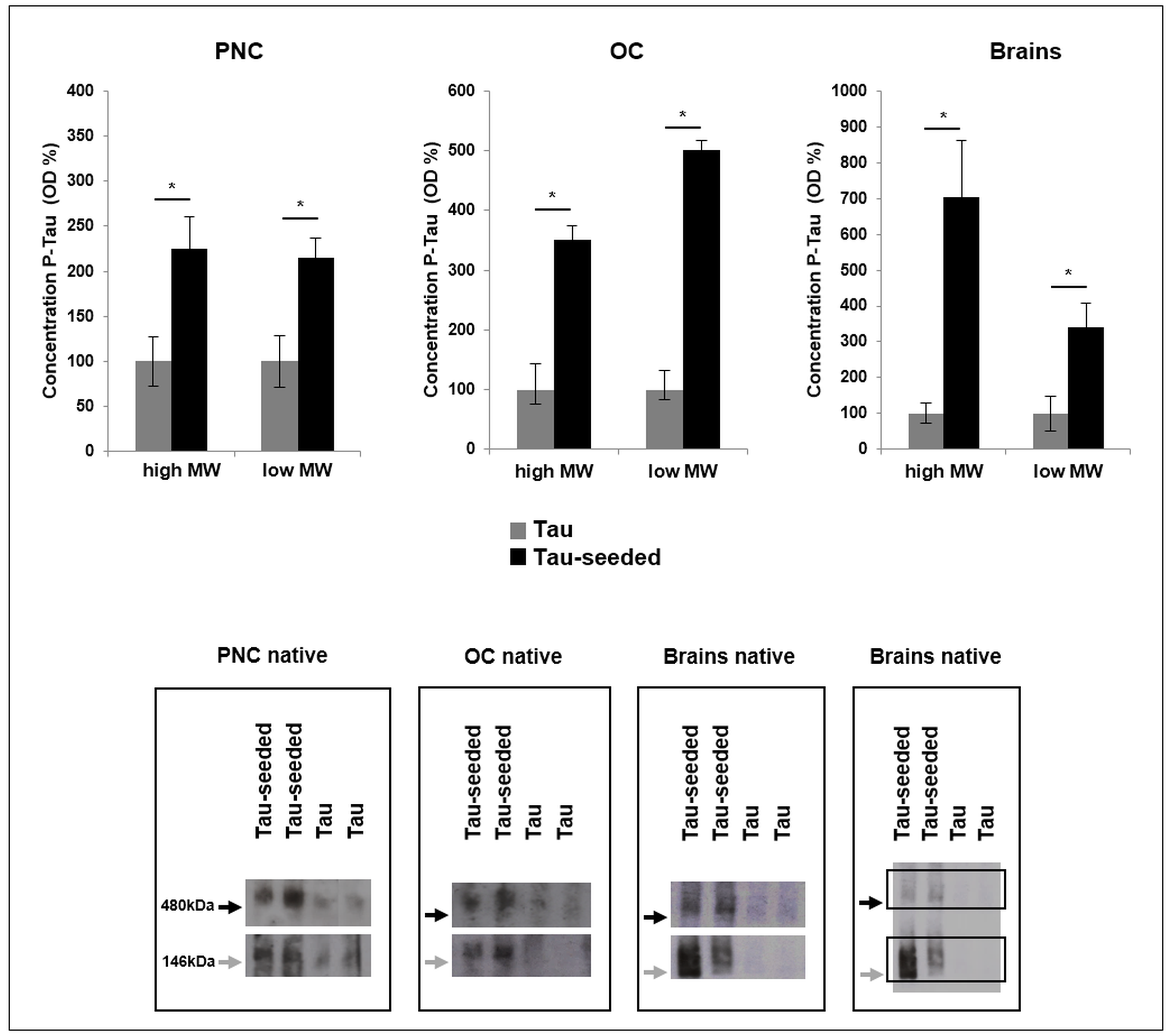

Supplement: Supplementary file 6 — Supplementary material 6 (TIFF 13614 kb) [file 401_2015_1413_MOESM6_ESM.tif]

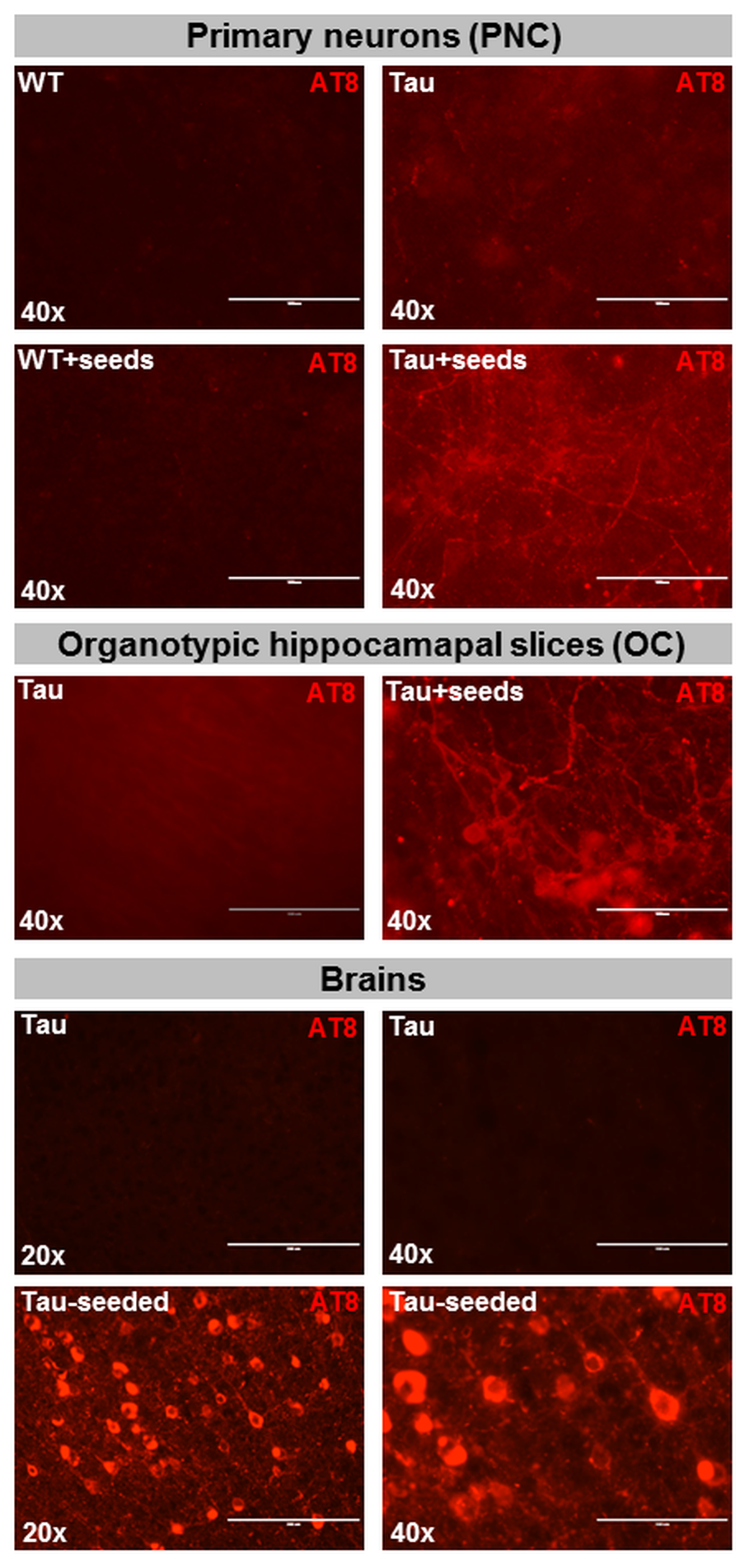

Supplement: Supplementary file 7 — Supplementary material 7 (TIFF 5042 kb) [file 401_2015_1413_MOESM7_ESM.tif]
