# Supplementary material for: Templated misfolding of Tau by prion-like seeding along neuronal connections impairs neuronal network function and associated behavioral outcomes in Tau transgenic mice
Source: Acta Neuropathol. 2015 Apr 11;129(6):875–94. doi: 10.1007/s00401-015-1413-4 (PMC4436846; doi:10.1007/s00401-015-1413-4)
Supplement: Supplementary file 8 — Supplementary material 8 (DOCX 58 kb) [file 401_2015_1413_MOESM8_ESM.docx]

Templated misfolding of Tau by prion-like seeding along neuronal connections impairs neuronal network function and associated behavioral outcomes in Tau transgenic mice

Acta Neuropathologica

Ilie-Cosmin Stancu^1*^, Bruno Vasconcelos^1*^, Laurence Ris^2^, Peng Wang^1^, Agnès Villers^2^, Eve Peeraer^3^, Arjan Buist^3^, Dick Terwel^4^, Peter Baatsen^5^, Tutu Oyelami^3^, Nathalie Pierrot^1^, Cindy Casteels^6^, Guy Bormans^6^, Pascal Kienlen-Campard^1^, Jean-Nöel Octave^1^, Diederik Moechars^3^, Ilse Dewachter^1#^

* contributed equally to this work

# corresponding author: Ilse.Dewachter@uclouvain.be, tel: (00 32) 2 764 93 36

1 Alzheimer Dementia Group, Institute of Neuroscience, Catholic University of Louvain, 1200 Brussels, Belgium

2 Department of Neurosciences, University of Mons, 7000 Mons, Belgium

3 Department of Neuroscience, Janssen Research and Development, A Division of Janssen Pharmaceutica NV, 2340 Beerse, Belgium

4 reMYND nv, Gaston Geenslaan 1, 3001 Leuven, Belgium

5 VIB11 vzw Center for the Biology of Disease, KU Leuven, 3000 Leuven, Belgium

6 MoSAIC - Molecular Small Animal Imaging Centre, KU Leuven, 3000 Leuven, Belgium

Supplemental Materials and methods

Animals

Transgenic TauP301S mice (PS19) [73] expressing the T34 isoform of microtubule-associated protein Tau with one N-terminal insert and four microtubule binding repeats (1N4R) encoding the human P301S mutation, driven by the mouse prion protein promoter were backcrossed to C57B6 and used in this study. TauP301S mice bred and used in our lab, display a less robust phenotype than previously reported, probably related to the background of the strain, genetic drift and maybe environmental factors [35, 63]. Neurofibrillary tangles and a neurodegenerative phenotype reflected in motoric deficits, clasping and development of hunchback starts at ~ 10-11 months of age. The animals were used for surgery at 3 months of age. Age-matched littermates were used for analysis. Genotyping was performed on tail biopsy DNA by polymerase chain reaction. Animals were housed on a 12 hours light/dark cycle in standard animal care facilities and acclimatized to the behavior facility prior to behavioral assessment, with access to food and water ad libitum. All experiments were performed in compliance with protocols approved by the UCLouvain Ethical Committee for Animal Welfare.

Generation of Tau-PFFs from recombinant Tau

Tau-PFFs or Tau-seeds were generated as described [26, 35]. Briefly, truncated human Tau fragments containing the four repeat domain (K18; residues Q244-E372 of the longest human Tau isoform) with a P301L mutation were produced in *Escherichia coli*. The fragments were flanked by a Myc-tag at their N- terminal end. To obtain Tau-PFFs, Tau fragments (66 μM) were incubated for 5 days at 37°C in the presence of 133 μM heparin (MW ~ 3 kDa; MP Biomedicals, Santa Ana, CA, USA) in 100 mM ammonium acetate buffer (pH 7.0). Afterwards, the fibrillization mixture was centrifuged at 100,000g for 1h at 4°C. The resulting pellet was resuspended in 100 mM ammonium acetate buffer (pH 7.0) at a final concentration of 333 µM Tau-PFFs and sonicated before use. Successful Tau fibrilization was confirmed using Thioflavin T (Sigma-Aldrich, St. Louis, MO, USA) fluorescent assay and western blotting analysis.

Tau aggregation assays

*In vitro Tau aggregation assay in HEK293 cells* HEK293 (QBI) cells were grown in full media (DMEM, 10 % FBS; Invitrogen, Life Technologies, Carlsbad, CA, USA) supplemented with penicillin/streptomycin (PenStrep). For fibril transduction, cells were plated at 1x10^5^ cells/well in 12-well tissue culture plates 1 day before transient transfection with P301L mutant Tau using FuGENE6 (Promega, Madison, WI, USA) at 0.4 μg of DNA per well. On the day of the fibrils transfection, Tau-PFFs were diluted to 10 μM in 100 mM ammonium acetate buffer (pH 7.0) and sonicated with 8 pulses. Of the resultant solution, 80 μL was added to the BioPORTER (AMS Biotechnology, Milton, UK) single use tubes and mixed. The tubes were vortexed at 1,000rpm and incubated 10 minutes at room temperature (RT) to form complexes. The cells were washed with OptiMEM (Invitrogen, Life Technologies, Carlsbad, CA, USA) and 500 μL OptiMEM was added to each well before the addition of 500 µL of fibril-reagent complexes. *In vitro Tau-aggregation assay in primary neurons* Primary cortical neuronal cultures (PNC) were generated as described previously [55], from P0 heterozygous TauP301S pups or non-transgenic littermates from a heterozygous crossing of TauP301S mice. Tau-seeds (10 nM) were added at DIV3 and DIV6, and primary neurons were used for calcium imaging at DIV13 and subsequently fixed for biochemical and ICC analysis. *Ex-vivo Tau-aggregation assay in organotypic hippocampal slices* Organotypic hippocampal slices (OC) were generated using previously described protocols [21, 64]. Briefly, hippocampal slices were generated from P6 heterozygous TauP301S pups and non-transgenic littermates. Tau-seeds (1 µL; 333 µM) were gently added on top of hippocampal slices at DIV3 and DIV6, and slices were analyzed electrophysiologically, biochemically and immunohistologically 10 days after seeding.

*Tau-seeded Tau-aggregation in vivo* For stereotactic surgery, three months old mice were deeply anesthetized with a mixture of ketamine/xylazine (Ketalar/Rompun). Unilateral (right hemisphere) or bilateral stereotactic injections were performed in the hippocampal CA1 region (A/P, -2.0 mm; L, + 1.4 mm; D/V, -1.2 mm) or frontal cortex (A/P, +2.0 mm; L, +1.4 mm; D/V, -1.0 mm). Injections in entorhinal cortex and substantia nigra were performed at (A/P, -4.8 mm; L, -3.0 mm; D/V, -3.7 mm) and (A/P, -4.8 mm; P/A, angle 16°; L, -1.1 mm; D/V, -4.7 mm) respectively, all coordinates are expressed relative to bregma [45]. Sonicated pre-aggregated Tau-PFFs (5 µL; 333 µM) or vehicle without seeds (5 µL) were injected using a 10 µL Hamilton syringe at a speed of 1 μL per min. After injection, the needle was kept in place for additional 5 min before gentle withdrawal.

Biochemical analysis

For immunoblotting analysis brains or selected brain regions were dissected and snap-frozen in liquid nitrogen. Total homogenates, sarkosyl soluble and sarkosyl insoluble fractions were prepared as described previously [68]. Briefly, after dissection, brain tissue was homogenized, with 10 strokes at 700 rpm on ice, in six volumes of Tris-NaCl buffer (25 mM TrisHCl, 150 mM NaCl, 1 mM EDTA, 1 mM EGTA, 5 mM Na_4_P_2_O_7_, pH 7.6) with addition of 1 mM PMSF, protease and phosphatase inhibitors cocktail (F. Hoffmann-La Roche AG, Basel, CH). Half of this total homogenate was stored at -80 °C and half was used for the sequential extraction, by ultracentrifugation at 150,000g for 30 min at 4 °C. The pellet was resuspended with equal volume of 10 mM TrisHCl, 0.8 M NaCl, 10 % sucrose, pH 7.6 with addition of 1 mM PMSF, protease and phosphatase inhibitors cocktail. Subsequently it was centrifuged at 20,000g for 30 min at 4 °C, the supernatant was incubated with 1 % sarkosyl (N-lauroylsarcosine sodium salt; Sigma-Aldrich, St. Louis, MO, USA) for 1 h at RT and ultracentifugated at 150,000g for 30 min at 4 °C. The supernatant (sarkosyl soluble fraction) and the resuspended pellet (sarkosyl insoluble fraction) were stored at -80 °C. Similar extraction procedures were used for preparation of total homogenates, sarkosyl soluble/insoluble fraction of HEK293 cells, primary neurons and organotypic cultures. For the separation of soluble and insoluble Tau using the Triton/SDS method, HEK293 cells were washed with phosphate buffered-saline (PBS), scraped into Triton lysis buffer [1 % Triton X-100 (TX-100), 50 mM Tris, 150 mM NaCl, pH 7.6] containing protease and phosphatase inhibitors cocktail and incubated on ice for 15 minutes. Cell extracts were sonicated and centrifuged at 100,000g for 30 minutes at 4 ⁰C. The supernatants yielded the Triton soluble fraction (soluble proteins), while the pellets were resuspended, sonicated and vortexed in SDS lysis buffer (1 % SDS, 50 mM Tris, 150 mM NaCl, pH 7.6) and stored as Triton insoluble fraction, after centrifugation at 100,000g for 30 minutes at 4 ⁰C. Proteins were quantified using BCA Protein Assay kit (Thermo Fisher Scientific, Waltham, MA, USA). For dot blotting equal amounts of total homogenates were spotted on a nitrocellulose membrane and subsequently immunoblotted using T22 [ABN454 Anti-Tau (T22) oligomeric antibody; EMD Millipore], against oligomeric Tau [39]. Analysis under non-denaturating and non-reducing conditions was performed on 4-16 % Bis-Tris Native Page. For immunoblotting equal amounts of proteins were loaded on precasted gels [4-12 % (MOPS), 8% Tris-glycine gel (Invitrogen, Life Technologies, Carlsbad, CA, USA)]. Immunoblotting was performed with anti-Tau P-S202/T205 (AT8; Thermo Fisher Scientific, Waltham, MA, USA), anti-Tau P-S396/S404 antibody (AD2; Bio-Rad Laboratories Inc., Hercules, CA, USA), and anti-total Tau antibody (HT7; Thermo Fisher Scientific, Waltham, MA, USA) and developed using ECL kit (PerkinElmer, Waltham, MA, USA). For AlphaLISA, HEK293 cells were seeded at 5x10^4^ cells per well in 24 multi-well plates (Falcon, LifeSciences) in DMEM (10 % FBS, 1 % PenStrep). The cells were transfected with WT Tau or P301L mutant Tau 24 hours later with FuGENE6 (Promega, Madison, WI, USA). The cells were re-seeded 24 hours after transfection in 384 multi-well plates at 3x10^3^ cells per well and 500 nM K18 fibrils was added. The cells were lysed in lysis buffer (1 % TX-100, 50 mM Tris, 150 mM NaCl, pH 7.6, supplemented with phosphatase and protease inhibitors) 2 days after the fibril treatment. Using AlphaLisa (Perkin Elmer) with an antibody generated in house (Johnson&Johnson) directed against a part of Tau outside the K18 seeds, Tau-aggregation levels were determined. This technique was validated by performing parallel immunoblotting of detergent insoluble fractions of HEK293 cells, demonstrating that aggregated Tau is detected.

Immunohistological and immunocytological analysis

Immunohistochemical analysis was performed as described previously [16, 17, 36, 49, 63]. Mice were transcardially flushed with ice-cold PBS for 2 minutes. Subsequently brains were dissected and immersion fixed in 4 % paraformaldehyde in PBS for 24 h at 4 °C for histological analysis. Sagittal sections (40 µm) were cut on a vibrating HM650V microtome (Thermo Fisher Scientific, Waltham, MA, USA). Immunohistochemistry with anti-Tau P-S202/T205 (AT8), anti-Tau P-S212/T214 (AT100; Thermo Fisher Scientific, Waltham, MA, USA) was performed on free-floating sagittal vibratome sections using appropriate Alexa coupled secondary antibodies (Invitrogen, Life Technologies, Carlsbad, CA, USA). Staining with Thioflavin S (ThioS; Sigma-Aldrich, St. Louis, MO, USA) and Gallyas silver (all chemicals from Sigma-Aldrich, St. Louis, MO, USA) staining were performed as previously described [63], and were used to demonstrate the “amyloid” nature of Tau aggregates in brain sections. Image acquisition was performed using a digital inverted fluorescence microscope EVOS-xl microscope (Life Technologies, Carlsbad, CA, USA), using a 4x, 10x, 20x or 40x lens. Gallyas silver staining was analyzed using a standard light microscope. Image analysis was done using Image J (National Institutes of Health). Heat maps were generated using the HeatMap Histogram plugin for Image J. Briefly the overview image of AT8 staining of a well-identified section of different mice (n ≥ 3 per group) were grouped. Stacked images representing averaged intensities were generated using the Image J stacking tool with the average intensities outcome option. Finally a Gaussian Blur filter of 5.0 was applied. ICC and IHC on HEK293 cells, primary neurons and organotypic cultures was performed similarly. Fixation was performed using standard fixation (4 % PFA) or under stringent extraction of soluble Tau using (4 % PFA, 1 % TX-100 or methanol). The latter fixation was demonstrated to extract soluble forms of Tau, as no signal was obtained following staining of Tau-expressing cells with a Total Tau antibody following stringent extraction. After fixation immunhisto/cytological staining was performed with anti-Tau P-S202/T205 antibody (AT8), anti-Tau P-T212/S214 antibody (AT100) and anti-conformational specific Tau (MC1, Peter Davies) using a standardized ICC protocol. All other chemicals were from Sigma-Aldrich (Sigma-Aldrich, St. Louis, MO, USA).

Neuronal network activity - spontaneous cytosolic calcium oscillations

Neurons derived from TauP301S transgenic mice and non-transgenic littermates were plated at a density of 2.3 x 10^5^ cells/cm^2^ on poly-L-lysine coated 22 mm round glass coverslips. For calcium imaging, neurons were incubated in the dark in the presence of the Ca^2+^ indicator fura-2 acetoxymethylester (Fura-2 AM; Calbiochem, Camarillo, CA, USA) at a final concentration of 2 μM in Krebs-HEPES buffer (10 mM HEPES, 135 mM NaCl, 6 mM KCl, 2 mM CaCl_2_, 1.2 mM MgCl_2_, 10 mM glucose, pH 7.4) for 30 min at RT. Coverslips were then washed in Krebs-HEPES buffer and mounted in a heated (37°C) microscope chamber with 600 µL of buffer [44, 55]. After recording of basal spontaneous calcium oscillations, picrotoxin (PTX; Sigma-Aldrich, St. Louis, MO, USA) was added to the neurons to a final concentration of 100 µM for 3 minutes. Cells were alternately excited (1 or 2 Hz) at 340 and 380 nm for 100 ms using a Lambda DG-4 Ultra High Speed Wavelength Switcher (Sutter Instrument, Novato, CA, USA) coupled to a Zeiss Axiovert 200 M inverted microscope (20x fluorescence objective) (Carl Zeiss AG, Oberkochen, DE). Images were acquired with a Zeiss Axiocam camera coupled to a 510 nm emission filter and analyzed with the AxioVision software. Calcium concentration was evaluated from the ratio of fluorescence emission intensities excited at the two wavelengths, i.e. the ratio of F340/F380. Changes in the intracellular calcium fluorescence were expressed as ΔF/F_0_ to represent the changes in the cytosolic calcium concentrations, where F is the cytosolic calcium fluorescence value at time t and F_0_ is the resting fluorescence value. Calcium oscillations were defined as variations of more than 10 % from F_0_, occurring synchronously in several cells of the field.

Functional imaging - FDG-PET analysis

Brain glucose metabolism was assessed using [^18^F]FDG (FDG). FDG was prepared with an Ion Beam Applications FDG synthesis module. Approximately 13 MBq (350 μCi; specific activity range, 100–760 GBq/μmol) of the radioligand was injected into the tail vein using an infusion needle set. Small-animal PET imaging was performed using a lutetium oxyorthosilicate detector-based tomograph (small-animal PET Focus 220; Siemens Medical Solutions, Erlangen, DE), which has a transaxial resolution of 1.4 mm in full width at half-maximum. Data were reconstructed in a 256 × 256 × 95 matrix with a pixel width of 0.316 mm and a slice thickness of 0.796 mm. The coincidence window width was set at 6 ns with an energy window of 350–650 keV. Before imaging, mice were anesthetized with 2.5 % isoflurane in 2.0 L/min oxygen. FDG measurements were obtained dynamically for 90 min. The acquisition timing rationale and kinetics of the radioligand in mice have been described previously [9]. For quantification purposes, PET scans were reconstructed using an iterative maximum a posteriori probability algorithm with ordered subsets (MAP; 18 iterations, 9 subsets, fixed resolution: 1.5 mm). Individual FDG-PET images were normalized to custom-made, ligand-specific mouse templates in Paxinos stereotactic space, allowing the use of a pre-defined Volume-of-interest (VOI) map, including the hippocampus, perirhinal – cerebral – and frontal cortex. The following methods of quantification were used: (i) standardized uptake values with and without correction for blood glucose levels, and (ii) normalizing counts to the whole-brain uptake.

Electrophysiology

*For electrophysiological analysis in organotypic hippocampal slices* Electrophysiological analysis was performed on non-seeded (vehicle) or Tau-seeded cultured hippocampal slices (at DIV3 and DIV6) and analyzed after 10 days of seeding. Culture inserts (MilliCell; Merck Millipore, Billerica, MA, USA) were directly placed into the recording chamber and slices were kept in interface at 28 °C for 30 min before recordings. *For electrophysiological analysis in acute hippocampal slices* Electrophysiological analysis was performed on hippocampal slices derived from Tau transgenic mice, 6 months post-seeding in entorhinal cortex, as described previously with slight modifications [17, 63]. Briefly, mice were anesthetized and decapitated, hippocampus was dissected and cut in 450 µm-thick slices with a tissue chopper. The slices were transferred into the recording chamber and kept in interface at 28 °C for 90 minutes. *Electrophysiological recordings* Hippocampal slices were perfused with artificial cerebrospinal fluid (ACSF) with the following composition: 124 mM NaCl, 5 mM KCl, 26 mM NaHCO_3_, 1.24 mM NaH_2_PO_4_, 2.5 mM CaCl_2_, 1.3 mM MgSO_4_, 10 mM glucose, bubbled with a mixture of 95 % O_2_ and 5 % CO_2_. The perfusion rate of ACSF was 1 mL/min. A bipolar twisted nickel-chrome electrode (50 µm diameter) was used to stimulate Schaffer’s collaterals. Extracellular field excitatory postsynaptic potentials (fEPSP) for acute slices or population spikes (PS) for organotypic slices were recorded in the stratum radiatum of the CA1 region with low resistance (2-5 MΩ) glass microelectrodes filled with ACSF. Test stimuli were biphasic (0.08 ms for each pulse) constant-voltage pulses delivered every minute with an intensity adjusted to evoke an approximate 40 % maximal response. The slope of the fEPSP or the amplitude of PS were measured on the average of four consecutive responses. LTP was induced by applying one train of high-frequency stimulation (100 Hz, 1 s). Stimulation, data acquisition and analysis were performed using the WinLTP program 28 (website: www.winltp.com). For each slice, the fEPSP slopes or PS amplitudes were normalized with respect to the mean slope of the fEPSPs or the mean amplitude of the PS recorded during the 30 minute period preceding induction of LTP. Statistical analysis of the data was performed using one-way ANOVA or Student’s t test in SigmaPlot 12.0 software (Systat Software Inc, Chicago, IL, USA). Data were expressed as means ± SEM, and differences with *p* < 0.05 were considered significant.

Behavioral testing

*The object recognition task* The object recognition task was essentially performed as previously described [17, 51]. Briefly, the mice were habituated for 10 min to a perspex open field box (60 × 60 × 50 cm). The next day the animals were placed in the same box and submitted to a 10 min acquisition trial. During this trial mice were placed in the open field in the presence of object A, and the time spent exploring object A (when the animal’s snout was directed toward the object at a distance < 1 cm) was measured. During a 10 min retention trial, which was performed 1 h later, a novel object (object B) was placed together with the familiar object (object A) in the open field. The time (tA and tB) the animal spent exploring the two objects was recorded. The recognition index (RI), defined as the ratio of the time spent exploring the novel object over the time spent exploring both objects [(tB/(tA + tB)) x 100] was used to measure non-spatial memory [17].

*Clasping scoring S*coring of clasping was performed using a scale between 5 and 1, with clasping score 5 representing no clasping (normal), score 4 representing initial signs of clasping, score 3 for intermediate clasping, score 2 for strong clasping, and score 1 representing very severe (maximal) clasping. Scores between 1 and 5 were assigned by an experimentator blinded for the experimental group. *Inverted grid hanging task* The inverted grid hanging test was used to assess the ability to grasp an elevated horizontal grid and to remain suspended for 2 minutes. The grid was positioned 50 cm above a soft, flat surface and measured 40 × 20 cm, with meshes with spacing of 0.5 cm. The latency for the animal to drop off was measured.

Immunogold Electron Microscopy

Proteins extracts from mouse brains, PNC, OC and HEK293 cells were applied on 300-mesh carbon-coated grids in drops of 3 µl for 5 min, blotted on a filter paper and air-dried before the immunogold procedure. Grids were blocked in PBS, 0.1 % cold water fish gelatin (PBS-CWFG) for 5 min before incubating with PBS-CWFG diluted primary antibodies (HT7 1:50, AT8 1:50, AT100 1:50) for 90 min at room temperature. After washing 5 times for 2 min in PBS-CWFG the grids were incubated for 60 min with secondary antibody (goat anti-mouse IgG) labeled with nanogold particles (Aurion, Wageningen, The Netherlands) diluted 1:30 in PBS-CWFG. Grids were washed 3 times for 2 min in PBS and 2 times for 2 min in dH2O and negatively stained with 2 % uranyl acetate for 1 min. The grids were examined with a JEOL JEM1400 transmission electron microscopy equipped with a Olympus SIS Quemesa 11 Mpxl camera, and images were taken at magnifications of 20x k and 30x k (resp. pixel size = 0.72 nm and 0.48 nm). Pre-embedding immune-electron microscopy of the Tau-injected mouse brains was performed on free-floating sagittal vibratome sections (40 µm) obtained as described above. The brain sections were postfixed for 15 minutes with 0.2 % glutaraldehyde in 0.1 M cacodylate buffer pH 7.4 and subsequently quenched with 0.1 % sodium borohydride in PBS for 10 minutes, blocked with 5 % horse serum, 0.1 % bovine serum albumin (BSA), 0.05 % TX-100 in PBS-CWFG for 30 minutes and then incubated with anti-Tau P-S202/T205 (AT8, 1:100) antibody in 0.1 % BSA and PBS-CWFG for 2 hours at room temperature. After washing 3 times for 5 min with PBS-CWFG, the brain sections were incubated for 60 minutes at RT with nano-gold labeled secondary antibody (goat anti-mouse IgG) (Aurion) diluted 1:30 in PBS-CWFG with 0.1 % BSA and 0.025 % TX-100 and subsequently washed 3 times for 5 minutes in PBS and fixed with 2.5 % glutaraldehyde in 0.1 M cacodylate buffer overnight at 4 °C. Sections were postfixed with 1 % osmium tetroxide for 1 hour at RT, dehydrated with graded series of ethanols at 4 °C, negatively stained with 3 % uranyl acetate at 4 °C for 30 minutes, and embedded in Agar100 resin at 60 °C for 48 hours. Ultrathin sections were cut and mounted on 300-mesh carbon-coated grids for subsequent EM analysis as described above.

Statistical analysis

Statistical analysis was performed using GraphPad Prism 5.0 (GraphPad Software, San Diego, CA, USA) using one-way analysis of variance (ANOVA) followed by Dunnett’s post hoc test and student’s t-test. Data were expressed as means ± SEM and differences were considered significant when *p*-values < 0.05.
