# Supplementary material for: Templated misfolding of Tau by prion-like seeding along neuronal connections impairs neuronal network function and associated behavioral outcomes in Tau transgenic mice
Source: Acta Neuropathol. 2015 Apr 11;129(6):875–94. doi: 10.1007/s00401-015-1413-4 (PMC4436846; doi:10.1007/s00401-015-1413-4)
Supplement: Supplementary file 9 — Supplementary material 9 (DOCX 22 kb) [file 401_2015_1413_MOESM9_ESM.docx]

Templated misfolding of Tau by prion-like seeding along neuronal connections impairs neuronal network function and associated behavioral outcomes in Tau transgenic mice

Acta Neuropathologica

Ilie-Cosmin Stancu^1*^, Bruno Vasconcelos^1*^, Laurence Ris^2^, Peng Wang^1^, Agnès Villers^2^, Eve Peeraer^3^, Arjan Buist^3^, Dick Terwel^4^, Peter Baatsen^5^, Tutu Oyelami^3^, Nathalie Pierrot^1^, Cindy Casteels^6^, Guy Bormans^6^, Pascal Kienlen-Campard^1^, Jean-Nöel Octave^1^, Diederik Moechars^3^, Ilse Dewachter^1#^

* contributed equally to this work

# corresponding author: Ilse.Dewachter@uclouvain.be, tel: (00 32) 2 764 93 36

1 Alzheimer Dementia Group, Institute of Neuroscience, Catholic University of Louvain, 1200 Brussels, Belgium

2 Department of Neurosciences, University of Mons, 7000 Mons, Belgium

3 Department of Neuroscience, Janssen Research and Development, A Division of Janssen Pharmaceutica NV, 2340 Beerse, Belgium

4 reMYND nv, Gaston Geenslaan 1, 3001 Leuven, Belgium

5 VIB11 vzw Center for the Biology of Disease, KU Leuven, 3000 Leuven, Belgium

6 MoSAIC - Molecular Small Animal Imaging Centre, KU Leuven, 3000 Leuven, Belgium

Supplemental figure legends

Figure S1 Synthetic Tau seeds induced Tau-aggregation in vitro: Efficiency of Tau-aggregation is increased with mutant TauP301 as recipient (and inducer) compared to WT Tau

*a*. Pre-aggregated synthetic Tau fragments referred to as Tau-seeds or Tau-PFFs, induce Tau-aggregation in vitro. TauP301L transient transfected HEK293 cells were transduced with Tau-seeds using BioPORTER reagent (Tau-seeded) or treated with BioPORTER alone (Tau). Abundant Tau aggregation was demonstrated using sequential extraction analysis. Total cell lysates, Triton soluble fraction, and Triton-insoluble fraction, following immunoblotting with AT8 are shown, demonstrating the presence of Triton-insoluble Tau following Tau-seeding. Similar results are obtained following sarkosyl extraction (results not shown) (*left panel*). Immunocytological analysis (*right panel*) demonstrates Tau-aggregation following Tau-seeding in TauP301L transiently transfected HEK293 cells. AT8 staining was used to reveal pathological Tau following Tau-seeding following stringent TX-100 extraction, absent in non-seeded Tau-expressing cells (*scale bar* 40 µm). *b*. Efficiency of Tau-aggregation is increased with mutant TauP301 as recipient (and inducer) compared to WT Tau. Alpha-Lisa analysis (*left panel*) demonstrating Tau aggregation efficiency using TauP301L and WT Tau both as recipient and inducer, demonstrates highest efficiency of Tau-aggregation for mutant TauP301L (*n* = 4 per condition; single ANOVA analysis, post Dunnett’s test between groups; t-test within group analysis; means ± SEM are presented; *p*-values ** < 0.01, *** < 0.001). Quantitation of ICC (*right panel*) of Tau-aggregation in HEK293 cells following seeding with TauP301L seeds in untransfected cells (Unt.), cells transfected with WT Tau or with TauP301L, demonstrates significantly increased AT8 staining in cells transfected with TauP301L compared to WT Tau and non-transfected cells. However Tau-aggregation is induced with WT Tau as recipient as well (*n* = 3 per condition; single ANOVA analysis post Dunnett’s test; means ± SEM are presented; *p*-values ** < 0.01, *** < 0.001). *c.* Immunocytological analysis of AT8 staining demonstrating difference in efficiency of Tau aggregation following seeding with TauP301L seeds (inducer) of WT Tau or P301 Tau (recipient), counterstaining with DAPI (*scale bar* 200 µm)

Figure S2 Analysis of spontaneous and picrotoxin induced calcium oscillations in primary neurons derived from non-transgenic mice following Tau-seeding

*a*. AT8 staining in primary neurons derived from non-transgenic mice following seeding (WT+Tau seeds; 10 days post-seeding) or without seeding (WT), in contrast to Tau-seed induced Tau aggregation (10 days post-seeding) in primary neurons derived from Tau transgenic mice (Tau-seeded). Representative stainings are shown revealing no AT8 staining in WT-seeded and WT-non-seeded primary neurons (*scale bar* 100 µm). *b*. Spontaneous (*grey bars*) and picrotoxin (*black bars*) induced calcium oscillations were not affected following seeding with Tau-seeds in primary neurons derived from non-transgenic mice. Calcium oscillations were measured using Fura-2 AM and expressed as ΔF/F_0_ (background corrected increase in fluorescence divided by the resting fluorescence F_0_) [WT (*n* = 26); WT+Tau-seeds (*n* = 24); experiments (*n* = 3)]. Calcium oscillations in the presence of picrotoxin were completely inhibited in the presence of CNQX and D-APV, indicating their AMPA and NMDA-dependence. Representative traces of calcium oscillations are presented (*right panels*) (representative for 3 independent experiments)

Figure S3 Analysis of pre- and post-synaptic markers following Tau-seeding in Tau P301S mice in EC

Parameters of presynaptic function were analyzed in acute hippocampal slices following initiation of Tau-aggregation in entorhinal cortex (EC) 6 months post-injection. Paired pulse ratio (PPR), a parameter of presynaptic function, was measured at different time intervals, revealing no significant changes between PPR measured in CA1 region of hippocampal slices derived from Tau-seeded or from non-seeded Tau mice [Tau-seeded (*n* = 5); Tau (*n* = 6); means ± SEM are presented; *p*-value < 0.05; student t-test] (*left panel*). Synaptic fatigue was measured as a parameter of presynaptic function, during high frequency stimulation used for induction of LTP. No significant differences were measured in CA1 region of hippocampal slices derived from Tau-seeded or non-seeded Tau mice [Tau-seeded (*n* = 5); Tau (*n* = 6)] (*right panel*)

Figure S4 Analysis of Tau-seeding in non-transgenic mice

Representative images of Tau seeding in non-transgenic (WT) mice, following injection in EC at 3.5 months and 12 months post-injection, demonstrate absence of marked Tau-seed induced Tau-aggregation. For comparison Tau-seed induced Tau-aggregation following initiation in EC at 3.5 months post-injection is presented (*right panels*). 20x magnifications of entorhinal cortex (EntCx) and hippocampal CA1 region are presented (*scale bar* 200 µm)

Figure S5 Analysis of Tau-aggregation using immuno-gold electron-microscopy

Immunogold electron microscopy was performed on brain sections of Tau transgenic mice following Tau-seeding, following staining with AT8. This revealed the presence of Tau-fibrils and non-fibrillar large Tau-aggregates. Immunogold labels decorate fibrillary Tau as well amorphous Tau aggregates (*left upper panels*). Immunogold labeling with AT8 followed by electron microscopy of sarkosyl insoluble fractions derived from the different models [brains (*right upper panels*), organotypic cultures (*lower left panels*), and primary neurons (*lower right panels*)] following Tau- seeding reveals the presence of fibrillary Tau in brains following Tau-seeding. These fibrils are stained with different Tau-antibodies AT8, AT100 and HT7. Similarly small Tau-aggregates ~ 20 nm are observed in addition to larger non-fibrillar Tau-aggregates, revealed by AT8, AT100 and HT7 staining. No labeling was observed following incubation with secondary antibody only, or in sarkosyl insoluble fractions of non-transgenic mice (not shown). Fibrillar Tau is furthermore detected in organotypic cultures and in HEK293 cells (not shown) following Tau-seeding. No fibrils were detected in sarkosyl insoluble fraction of primary neurons, in contrast to readily observed small Tau-aggregates/oligomers (~ 20 nm) (*scale bar* 100 nm).

Figure S6 Biochemical analysis of pathological Tau-forms induced by Tau-seeding

Biochemical analysis of Tau in total homogenates under non-denaturing and non-reducing conditions was performed. Tau present in larger Tau-aggregates, including large multimers, granular Tau and fibrils cannot enter the gel under these conditions. Immunoblotting with AT8 of total homogenates on native page revealed Tau multimers ranging between 480-720 kDa and between 146-242 kDa, in line with the presence of multimers ≤ 10, and di- or trimers, respectively (*black arrow* indicates 480 kDa marker, *grey arrow* indicates 146 kDa marker). An overview blot showing high and low MW multimers in brain extracts is presented (*lower right panel*). The presence of these forms are in line with the elution profile of smaller (not granular aggregates) forms of toxic Tau-multimers in size exclusion chromatography that correlated with decreased memory index [3] in 6.5 months old rTG4510 mice. Different conditions for immuno-detection were used for optimal detection and quantitation of each Tau form (high MW Tau forms and low MW Tau forms respectively) in the different models. Representative blots of high MW and low MW forms in primary neurons (PNC), organotypic cultures (OC) and brains are presented. Quantitative analysis and comparison was performed between Tau forms detected under identical conditions, more particularly between Tau-seeded and non-seeded conditions, for low MW Tau and high MW Tau forms respectively, in each model. We here demonstrate that Tau-seeding increases the concentrations of AT8 positive Tau-multimers analyzed by native page immunoblotting. Quantitative analysis demonstrated accumulation of AT8 positive Tau multimers under non-reducing, non-denaturing conditions following Tau-seeding in brains, organotypic cultures (OC) and primary neurons (PNC) [*n* = 4 (Tau-seeded), *n* = 4 (non-seeded) for each condition; means ± SEM are presented; **p*-value < 0.05]

Figure S7 Tau-seed induced pathological forms of Tau

In PNC, AT8 staining after standard fixation in 4% PAF - omitting extraction of soluble Tau - demonstrates strong diffuse and punctated AT8 staining present throughout the culture dish, and robustly induced following Tau-seeding in TauP301S expressing PNC. Representative AT8 staining in Tau-seeded Tau expressing neurons (Tau-seeded), in non-seeded Tau-expressing neurons (Tau), in non-seeded (WT) and seeded wild-type neurons (WT-seeded) are presented. Immunohistological analysis with AT8 reveals different forms of AT8 stained Tau in brains and organotypic cultures following Tau-seeding. In addition to mature and immature NFTs, diffuse and punctated AT8 staining is strongly induced in organotypic hippocampal cultures and brains following Tau-seeding
